# Genome-wide metabolite quantitative trait loci analysis (mQTL) in red blood cells from volunteer blood donors

**DOI:** 10.1016/j.jbc.2022.102706

**Published:** 2022-11-15

**Authors:** Amy Moore, Michael P. Busch, Karolina Dziewulska, Richard O. Francis, Eldad A. Hod, James C. Zimring, Angelo D’Alessandro, Grier P. Page

**Affiliations:** 1 Division of Biostatistics and Epidemiology, RTI International, Atlanta, Georgia, USA; 2 Vitalant Research Institute, San Francisco, California, USA; 3 Department of Pathology, University of Virginia School of Medicine, Charlottesville, Virginia, USA; 4 Department of Pathology and Cell Biology, Columbia University Medical Center, New York, New York, USA; 5 Department of Biochemistry and Molecular Genetics, University of Colorado Denver – Anschutz Medical Campus, Aurora, CO, USA

**Keywords:** metabolomics, transfusion medicine, red blood cell, SNP, glucose 6-phosphate dehydrogenase, GAPDH, glyceraldehyde 3-phosphate dehydrogenase, G6PD, glucose 6-phosphate dehydrogenase, GWA, genome-wide association, LD, linkage disequilibrium, LPS, lysophospholipids, mQTL, metabolite quantitative trait loci, PPP, pentose phosphate pathway, RBC, red blood cell, REDS-III, Recipient Epidemiology and Donor Evaluation Study, SAM, S-adenosyl-methionine, SPTA1, spectrin alpha 1

## Abstract

The red blood cell (RBC)-Omics study, part of the larger NHLBI-funded Recipient Epidemiology and Donor Evaluation Study (REDS-III), aims to understand the genetic contribution to blood donor RBC characteristics. Previous work identified donor demographic, behavioral, genetic, and metabolic underpinnings to blood donation, storage, and (to a lesser extent) transfusion outcomes, but none have yet linked the genetic and metabolic bodies of work. We performed a genome-wide association (GWA) analysis using RBC-Omics study participants with generated untargeted metabolomics data to identify metabolite quantitative trait loci in RBCs. We performed GWA analyses of 382 metabolites in 243 individuals imputed using the 1000 Genomes Project phase 3 all-ancestry reference panel. Analyses were conducted using ProbABEL and adjusted for sex, age, donation center, number of whole blood donations in the past 2 years, and first 10 principal components of ancestry. Our results identified 423 independent genetic loci associated with 132 metabolites (*p* < 5×10^–8^). Potentially novel locus-metabolite associations were identified for the region encoding heme transporter FLVCR1 and choline and for lysophosphatidylcholine acetyltransferase LPCAT3 and lysophosphatidylserine 16.0, 18.0, 18.1, and 18.2; these associations are supported by published rare disease and mouse studies. We also confirmed previous metabolite GWA results for associations, including N(6)-methyl-L-lysine and protein PYROXD2 and various carnitines and transporter SLC22A16. Association between pyruvate levels and G6PD polymorphisms was validated in an independent cohort and novel murine models of G6PD deficiency (African and Mediterranean variants). We demonstrate that it is possible to perform metabolomics-scale GWA analyses with a modest, trans-ancestry sample size.

Red blood cells (RBCs) are the most abundant cell in the human body, representing approximately 84% of the ∼30 trillion host cells in an adult individual (1). Despite the lack of nuclei and organelles, RBCs are endowed with around 3000 proteins (2, 3, 4) that allow them to take up and metabolize gases (O_2_, CO_2_) and small molecule substrates. This task is facilitated by their transit through the whole body every ∼20 s, during the average 120 days lifespan of RBCs (5). From this perspective, RBC metabolism has been leveraged in clinical chemistry assays as a window on systems metabolism and dysregulation thereof (6, 7). The recent implementation of cost-effective high-throughput mass spectrometry-based metabolomics (8) has fueled the efforts toward personalized medicine.

RBC transfusion is a life-saving intervention for 4.5 million Americans every year. The logistics of producing over 100 million units of blood available for transfusion every year around the world necessitates storage of RBC components in the blood bank. However, storage in the blood bank is characterized by a series of biochemical (9) and morphological alterations (10), collectively referred to as the storage lesion (11), which ultimately lower the efficacy of the transfusion therapy (*e.g.*, hemoglobin increment upon transfusion) (12). Alterations to RBC energy and redox metabolism are a hallmark of the storage lesion (13, 14). Metabolic markers of RBC storage quality have been identified, including markers of the RBC propensity to hemolyze spontaneously (15) or following oxidant or osmotic insults (16), markers of the propensity of end of storage RBCs to circulate at 24 h upon transfusion (17, 18), and markers of oxygen transport and off-loading function in fresh and stored RBCs (19). Appreciation for the role of RBC metabolism in storage quality and post-transfusion performances has informed the concept of the metabolic age of the unit—as opposed to the chronological age of the unit (*i.e*., days elapsed since the time of donation) (20). Levels of metabolic markers of RBC storage quality and function are impacted by blood processing, storage additives, donor demographics (*e.g.,* sex, race-ethnicity, and age), dietary metabolites (21) or environmental factors/donor habits (*e.g.*, smoking, alcohol use, and drugs) (22, 23, 24, 25), donor age (26) and body mass index (27), and—relevant to the present study—donor genetics (28). In a move toward personalized transfusion medicine, the Recipient Epidemiology and Donor Evaluation Study—REDS-III RBC-Omics—was designed to test the hypothesis that donor biology plays a significant role in the quality of donated RBC (29, 30). As part of this study, genomics approaches were used to characterize 12,353 volunteer donors enrolled at four different blood centers across the United States of America (29, 30). Genetic heterogeneity of blood donors was associated with the RBC propensity to hemolyze spontaneously, or following oxidative, osmotic or mechanical insults. Quantitative trait-loci analyses were used to identify polymorphic genes that contribute to an increased resistance/susceptibility to RBC hemolysis (28). Of note, polymorphisms associated with a compromised (<10%) residual activity of glucose 6-phosphate dehydrogenase (G6PD) were associated with an increased susceptibility of stored RBCs to lyse following oxidant insults (28). This observation was corroborated by findings from *ex vivo* studies (31) and *in vivo* determination of autologous post-transfusion recovery (17), as well as decreases in hemoglobin increments upon transfusion of G6PD-deficient units (32). G6PD is the rate-limiting enzyme of the pentose phosphate pathway, the main antioxidant pathway in RBCs. Thus, G6PD is crucial for the synthesis of the reducing cofactor NADPH, which is required to preserve glutathione homeostasis and reduce multiple antioxidant enzymes as they exert their catalytic activities (33). G6PD deficiency is common in routine blood donors of African descent (up to 13% prevalence in some metropolitan areas like New York) (34). In G6PD-deficient donors, higher levels of metabolic markers of the storage lesion have been reported, such as a faster rate of purine deamination (35), asparagine deamidation and methylation (36), and lipid oxidation (16). However, to date, linkage of genetic polymorphisms to metabolic heterogeneity in freshly donated or end of storage blood has been limited to twin studies (15).

Blood donors are a selected population, with the basic requirement for volunteer blood donation being the absence of serious underlying medical conditions, adequate hemoglobin levels, absence of risk factors for transfusion-transmitted infections, and not taking specific teratogenic or other medications (22) that are grounds for deferral. As such, epidemiology studies have been facilitated by the study of large cohorts of blood donors, similar to investigations on SARS-CoV-2 incidence in the general population based on serological characterization of routine blood donors (37). Here, we leveraged genomic (28) and metabolomic data (29) generated as part of the REDS-III RBC-Omics study to perform a metabolite quantitative trait loci (mQTL) analysis of routine blood donors. The study builds on previous mQTL reports in the context of cardiovascular diseases, asthma, or neurodegenerative diseases, including Alzheimer’s disease (38, 39, 40, 41, 42). However, most of these studies focused on plasma, urine and cerebrospinal fluid, with limited analyses targeting erythrocyte metabolism—which is the focus of this study. Given the importance of RBC metabolism as a window into systems homeostasis, findings reported here could be relevant not just for transfusion medicine research but also for diverse areas of physiology where RBC metabolism is impaired (*e.g.*, exercise (43), aging (44), adaptation to high-altitude hypoxia (45), pathological hypoxia upon hemorrhage (46), COVID-19 (47, 48), cardiovascular (49) and kidney diseases (50, 51)).

## Results

**Figure 1:**
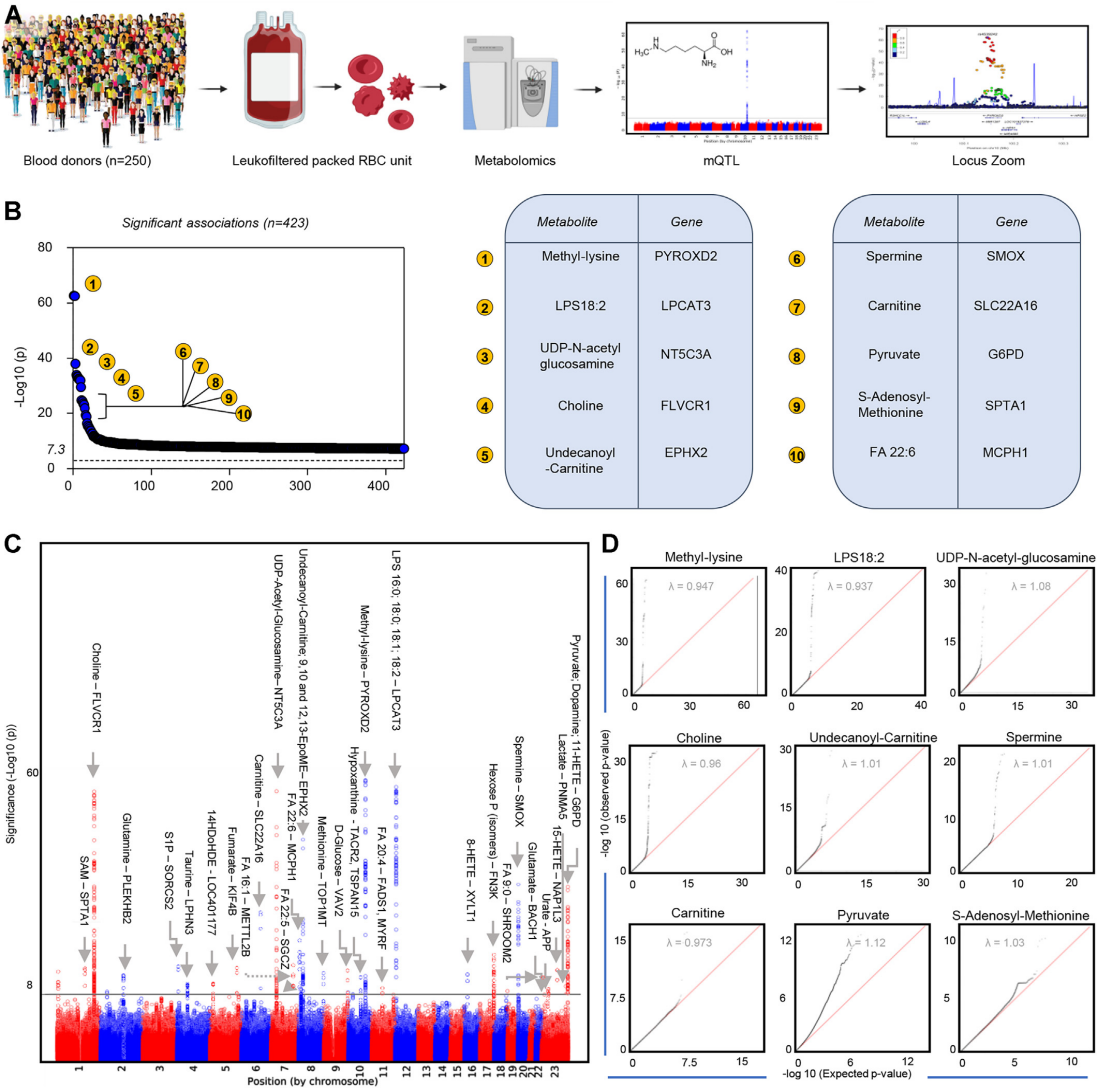
**Study design and top 10 hits from the mQTL analysis from the REDS-III RBC-Omics pilot recalled donor study.***A*, metabolomics analyses were performed on 250 packed RBC samples from donors who had been previously characterized at the genome level *via* the precision transfusion medicine array (30). *B*, an overview of the top 10 hits (closest annotated gene to the identified SNP) as a function of significance (-log10(p)). *C*, overlapped Manhattan plots of all the significant hits (FDR < 5 × 10^-8^), including metabolites—gene pairs. Each data point corresponds to a –log10(*p* value) from a multivariant linear regression model’s *p* value for an SNP. The *black horizontal line* represents an accepted *p*-value level of genome-wide significance (*p* = 5 × 10^–8^). *D*, Q-Q plots for the top 10 hits from the mQTL analysis. mQTL, metabolite quantitative trait loci; REDS-III, Recipient Epidemiology and Donor Evaluation Study.

**Figure 2:**
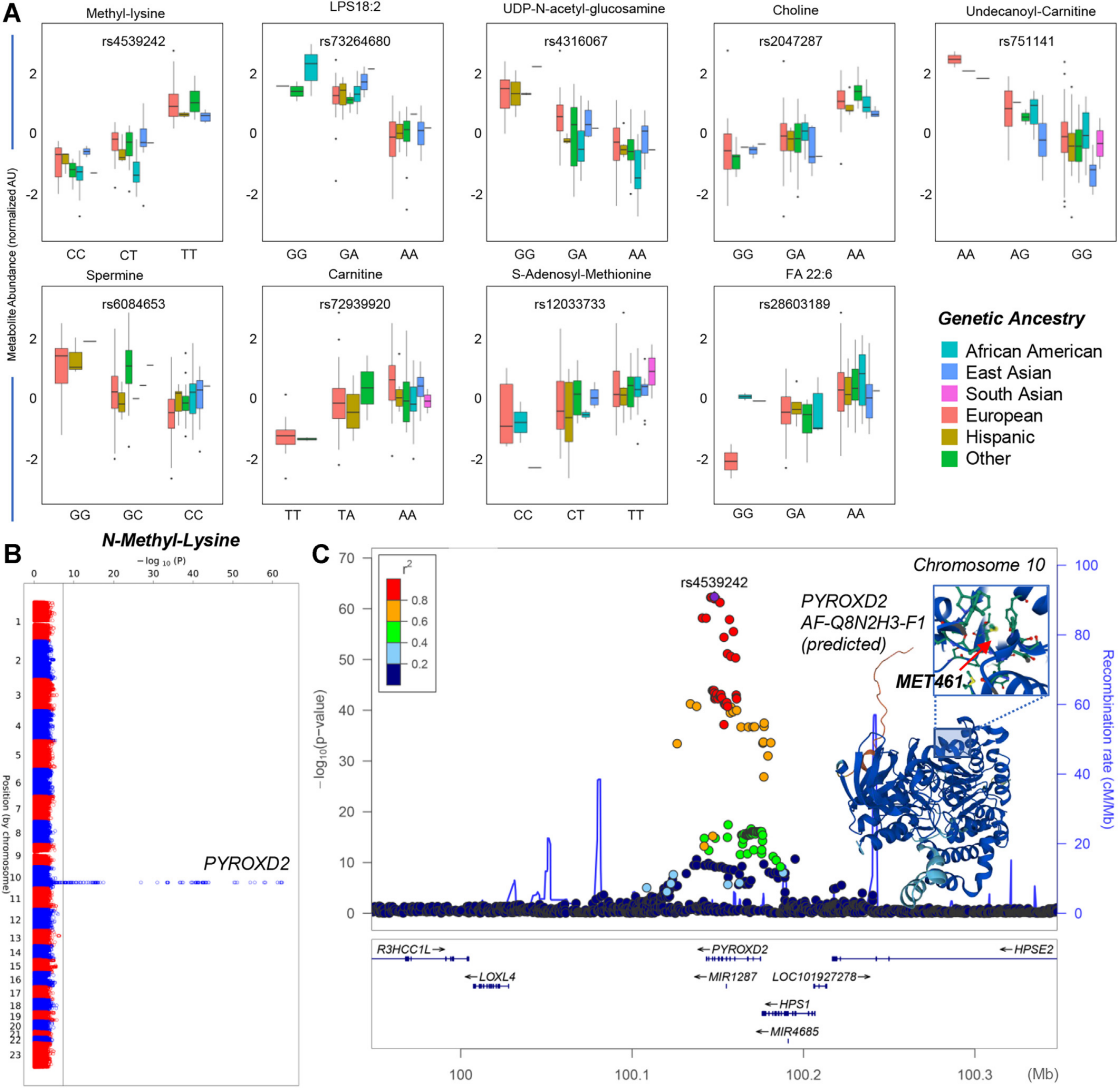
**Ancestry plots and association between methyl-lysine levels and polymorphisms in PYROXD2.***A*, for the top GWA hits we generated box and whisker plots based on metabolite abundances as a function of allele variance across all genetic ancestries in this study. Consistently with previous mQTL studies (40, 41, 65, 66, 67), polymorphisms in the exonic region coding for the enzyme PYROXD2 were associated with variance in the levels of methyl-lysine, an observation that represents a sort of internal quality control for the present analysis compared to the literature. *B–C*, Manhattan plots and LocusZoom are shown in panels *B*–*C*, respectively. GWA, genome-wide association.

Metabolomics analyses were performed on packed RBC samples derived from stored RBC components from 250 donors who had been previously characterized at the genome level *via* the Precision Transfusion Medicine array (Fig. 1*A*) (30). Through the workflow summarized in Fig. S1, a total of 2831 single nucleotide polymorphism (SNP)-metabolite associations were observed below the genome-wide correction threshold (*p* < 5.0 × 10^−8^). Data are summarized in tabulated form in Table S1 by identifying the SNP with the smallest *p*-value within a ± 500 kilobase range as the lead SNP; individual SNP associations are reported extensively in Table S1. In Fig. 1*B*, we listed the top 10 hits identified by closest annotated gene to the significant SNP in order of -log10(p). Manhattan plots overlapping all the significant hits (FDR < 5 × 10^-8^) are shown in Fig. 1*C*, which also includes metabolite—gene pairs. Q-Q plots for the top nine metabolite-associated SNPs are shown in Fig. 1*D*. Sensitivity analyses for 46 metabolites examining the impact of (1) more stringent variant quality control (QC); (2) the choice of imputation strategy for missing metabolite data; (3) the effect of blood storage additive; and (4) ancestry are reported in Table S1. Genetic associations identified for six metabolites have been previously reported (Table S1). Ancestry plots were thus generated to show normalized metabolite abundances as a function of alleles, as distributed across genetic ancestries of the donors enrolled in this study (Fig. 2*A*). We further characterized the mQTL loci by generating LocusZoom plots to examine the local linkage disequilibrium (LD) structure and performed *in silico* functional annotation using OASIS.

Consistent with previous mQTL studies(citations 40,41,64–67), the top SNP associated with levels of methyl-lysine, rs4539242, is in high LD with both the missense mutation M461T (R^2^ = 1.0 in Europeans) and synonymous mutation F484F (R^2^ = 0.94 in Europeans), observations that represent an internal quality control for the present analysis (Manhattan plots and LocusZoom in Fig. 2, *B* and *C*, respectively). Both mutations are themselves associated with levels of methyl-lysine (*p* = 4.22 × 10^−13^ and *p* = 1.28 × 10^−44^, respectively; Table S1).

**Figure 3:**
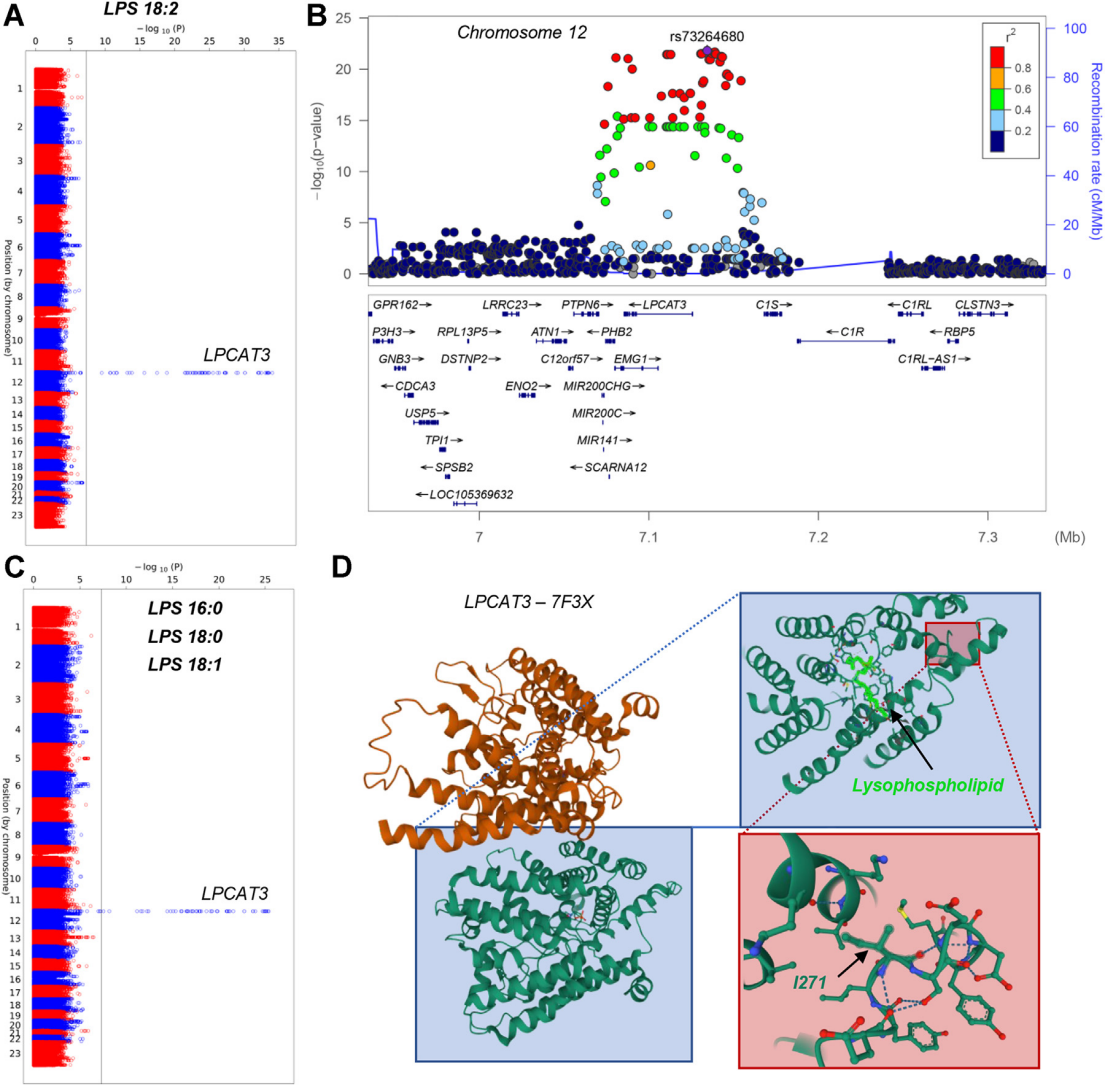
**LPCAT3 is polymorphic in healthy blood donors and associates with red blood cell lysophospholipid (LPS) levels.***A*–*D*, Manhattan plots for LPS, specifically linoleyl- (18:2—**A** and related LocusZoom, highlighting the association with the region coding for LPCAT3 in *B*), palmitoyl (16:0), stearoyl (18:0) or oleyl (18:1—***C***). *D*, highlight of the polymorphic residue I271, mapped against the structure of LPCAT3 (7F3X.pdb).

The region coding for the enzyme lysophosphatidylserine acetyltransferase 5 (LPCAT3) was found to be genetically heterogenous across volunteer blood donors. Polymorphisms in the region coding for LPCAT3 were the lead SNP, rs73264680, associated with RBC levels of lysophospholipids (LPSs), including linoleyl- (18:2), palmitoyl (16:0), stearoyl (18:0), or oleyl-LPS (18:1), rs73264680, is in perfect LD in Europeans with the missense mutation rs1984564/I217T within *LPCAT3* (Fig. 3, *A–C* and Table S1; residue mapped against the structure of LPCAT3—7F3X.pdb in Fig. 3*D*).

**Figure 4:**
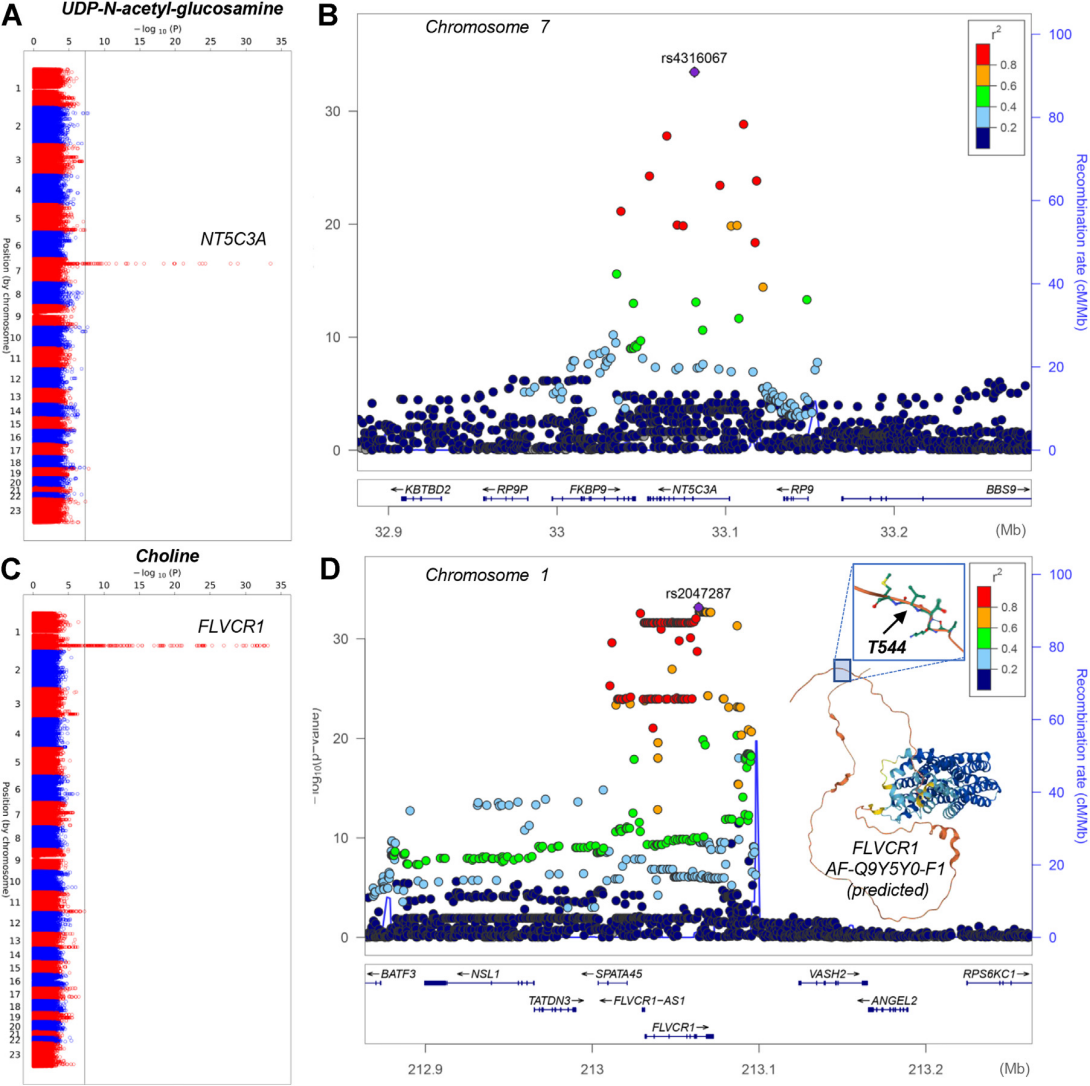
**Polymorphisms in NT5C3A and FLVCR1 are associated with variability in the levels of UDP-N-acetyl-glucosamine and choline in RBCs from healthy blood donors.***A*–*D*, Manhattan plots and LocusZoom are shown in panels A–B and C–D, respectively.

The lead SNP associated with UDP N acetyl glucosamine, rs4316067 (Table S1), is located in an intron of *NT5C3A* (Fig. 4, *A* and *B*). The lead SNP associated with choline, rs2047287 (Fig. 4, *C* and *D*), is in strong LD (D’=1.0; R2=0.768 in Europeans) with the missense mutation T544M in *FLVCR1*.

**Figure 5:**
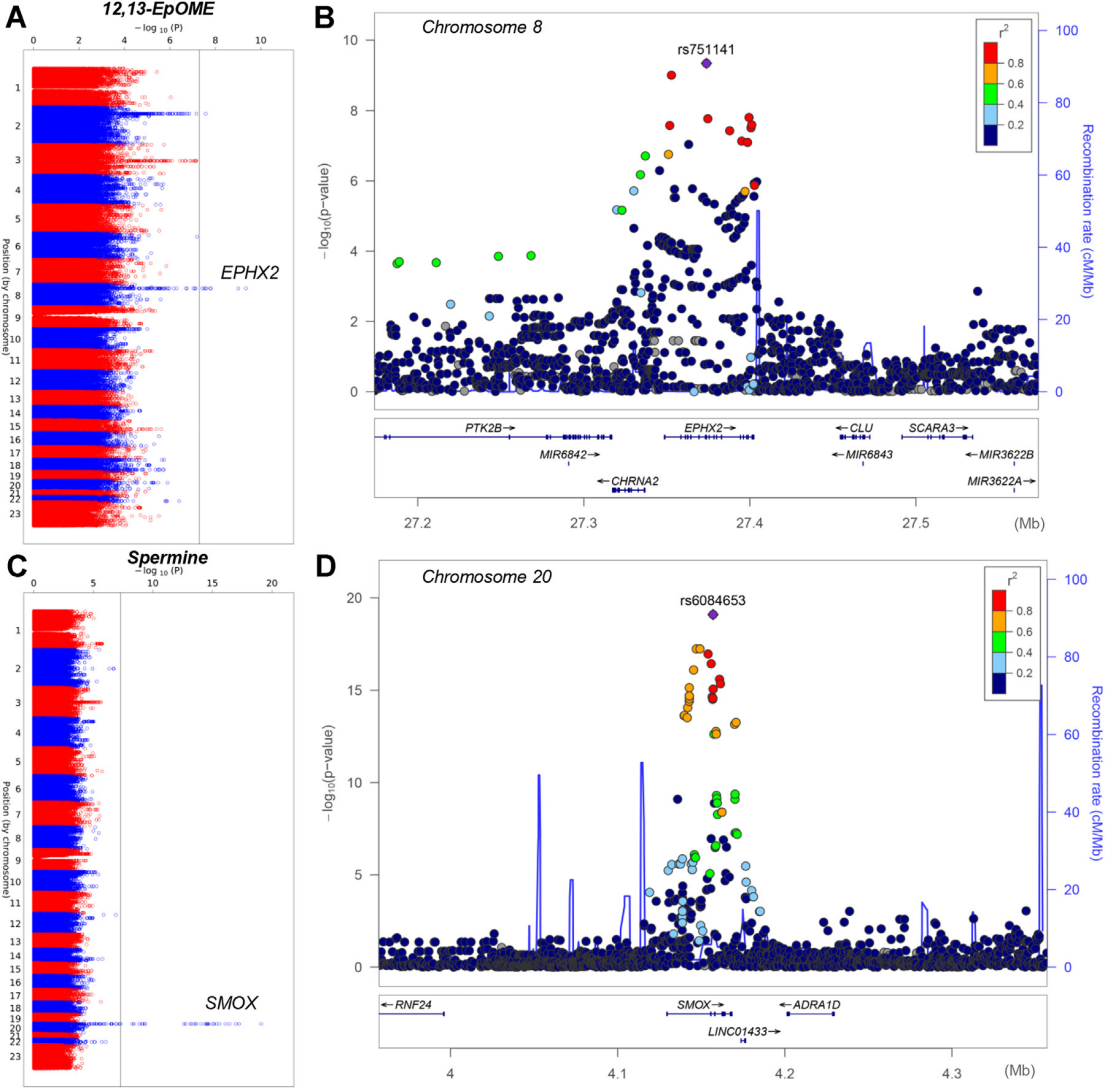
**Polymorphisms in EPHX2 and SMOX are associated with variability in the levels of oxylipins (12,13-EpOME) and spermine, respectively.***A–D*, Manhattan plots and LocusZoom are showns in panels *A* and *B* and *C* and *D*, respectively. EPHX2, epoxide hydrolase 2; SMOX, spermine oxidase.

Polymorphisms in bifunctional epoxide hydrolase 2 (*EPHX2—*missense mutation rs751141/R221Q—*p* = 4.55 10^-10^; λ = 1.01) and spermine oxidase, where the intronic rs11087622 in *SMOX—*is in LD (R^2^ = 0.16; D’=0.71 in Europeans) with synonymous mutation A392A (Table S1), are associated with variability in the levels of oxylipins (12,13-EpOME) and spermine, respectively (Fig. 5).

A missense variant (rs12210538/M409T) in the carnitine transporter *SLC22A16* is associated with variability in the levels of RBC free and acetyl-carnitine (Table S1). Manhattan plots and LocusZoom are shown in Fig. S2. Additional SNPs associated with carnitine levels include palmitoyl-carnitine (nearest gene *HTR5A-AS1*) and undecanoyl-carnitine (nearest gene *EPHX2*).

A series of significant associations were identified between the levels of oxylipins like 9.10-EpOME (EPHX2—Uniprot names provided for protein products of the nearest gene within parentheses in this paragraph) or 14-DHoHE (LOC401177 or LOC100505817) or 9-HETE, 15-HETE (NAP1L3), dopamine (G6PD), glycolytic metabolites (glucose and VAV2, hexose phosphate, including fructose 6-phosphate and FN3K; lactate and PNMA5), purines (hypoxanthine and TACR2, TSPAN15; urate and FOLR1 and APP), amino acids (glutamine and PLEKHB2; glutamate and BACH1-IT2; methionine and mitochondrial topoisomerase I; taurine and LPHN3, threonine and mR8058), free fatty acids (palmitoleic acid and METTL2B; oleic acid; arachidonic acid and FADS1; docosapentaenoic acid and SGCZ), sphingolipids (sphingosine 1-phosphate and EDARADD or SORCS2 or KDM6A), and uridine diphosphate (UDP and ZNF485—Figs. S3–S15).

**Figure 6:**
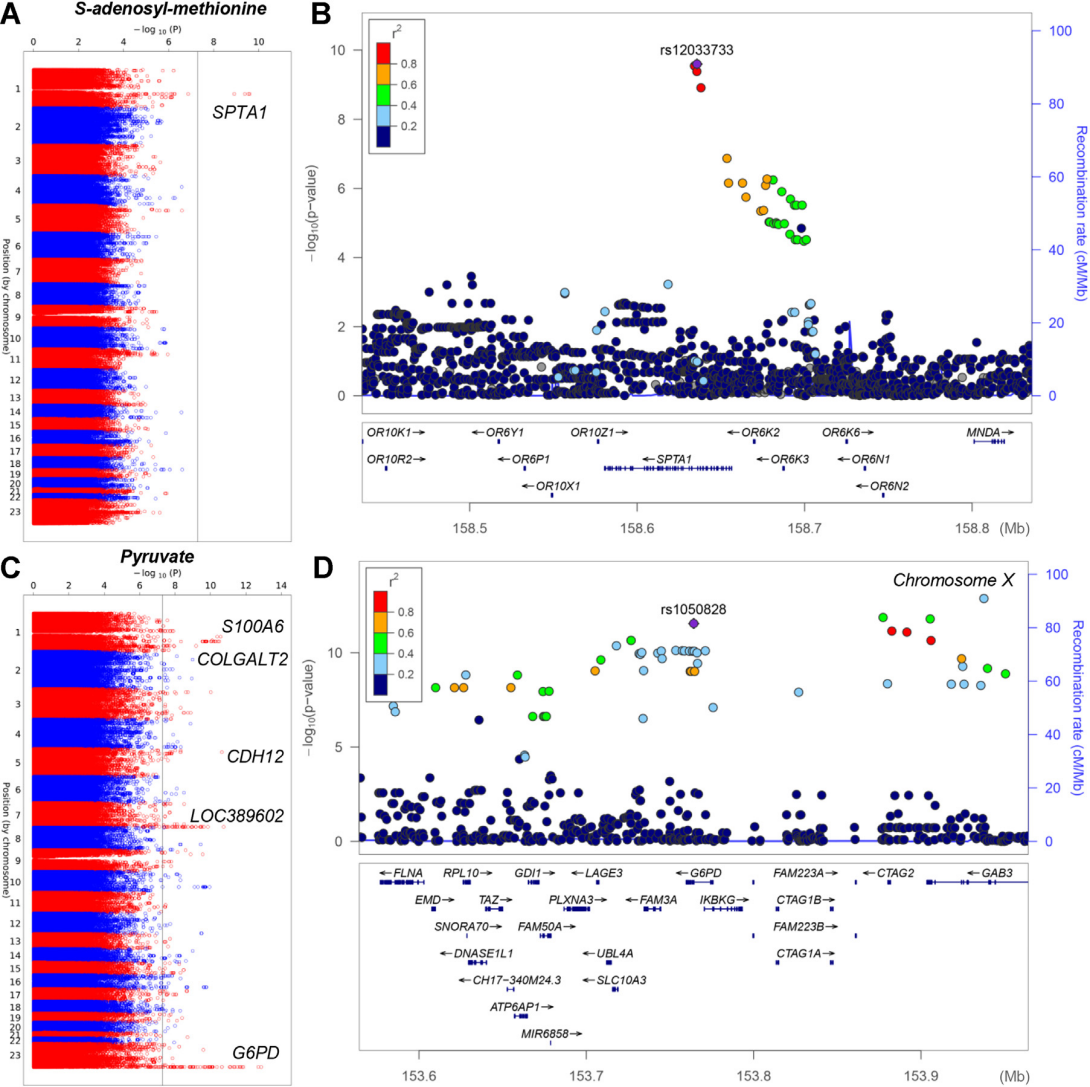
**Polymorphisms in SPTA1 and G6PD are associated with variability in the levels of S-adenosyl-methionine and pyruvate, respectively.** Manhattan plots and LocusZoom are shown in panels *A* and *B* and and *D*, respectively. SPTA1, spectrin alpha 1; G6PD, glucose 6-phosphate dehydrogenase.

Finally, polymorphisms in spectrin alpha 1 (*SPTA1)* and *G6PD* are associated with variability in the levels of S-adenosyl-methionine (SAM) (intronic - *p* = 2.52 10^−10^; λ = 1.03; Table S1) and pyruvate **μM** (missense mutation V98M - *p* = 2.87 10^−12^; λ = 1.12; Table S1), respectively (Fig. 6, *A*–*D* for Manhattan plots and LocusZoom).

### Sensitivity and replication analyses

We performed several sensitivity and analyses, including Genome-Wide Association Study (GWAS) of 46 metabolites in 176 study participants with available metabolomics data generated from RBC samples stored for 42 days, as replication of findings in fresh blood from the same subjects. The top associations were replicated for N6-methyl-L-lysine, LPS16.0-18.2, UDP N-acetyl-glucosamine, choline, undecanoyl carnitine, spermine, spermine uM, and L-carnitine. These findings replicated in each of the five sensitivity analysis, with either the original lead SNP or a genome-wide significant SNP in high LD with the original reaching genome-wide significance (Table S1). For docosahexaenoic acid (FA22.6), the association with rs28603189 replicated when stringent QC criteria were applied, when a different missing metabolite imputation strategy was employed, and when analysis was restricted to the participants whose RBC samples were stored in Additive 3 (R^2^ = 0.94), but not in the day 42 storage samples or the European-only analysis. The association between rs12033733 and S-adenosyl-L-methionine withstood the stringent QC but none of the other sensitivity analyses. Finally, although there were many genome-wide significant associations with pyruvate uM, the lambda was 1.115 and the Q-Q plot troubling (Fig. 1), potentially indicating unaccounted for population structure for this metabolite. The association between pyruvate uM and rs142516556, a SNP near the *G6PD* gene, remained robust to the stringent QC, imputation method, and Additive 3-restricted GWAS (Table S1).

### Elevated pyruvate and pyruvate/lactate ratios are recapitulated in an independent human cohort of blood donors and mouse models of G6PD deficiency

**Figure 7:**
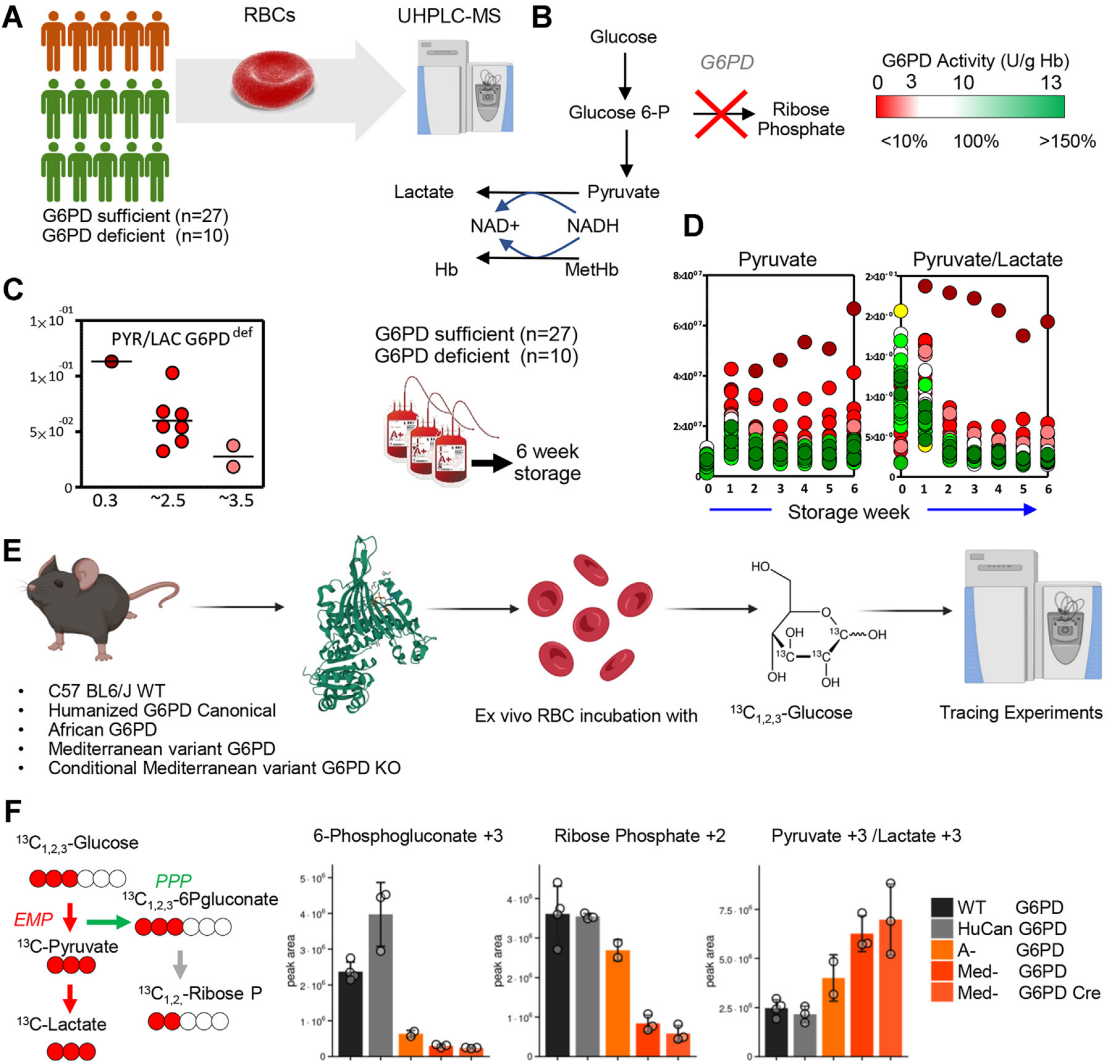
**G6PD deficiency in fresh and stored RBCs from blood donors are associated with increases in pyruvate levels and pyruvate/lactate ratios.***A–C*, pyruvate levels were found to be inversely proportional to G6PD activity in fresh RBCs from G6PD-deficient (n = 10) and -sufficient (n = 27) blood donors. *D*, these differences in pyruvate levels between the two groups were exacerbated during storage in the blood bank up to 42 days. *E*, similarly, RBCs from G6PD-deficient mice (African and Mediterranean variant) and WT C57BL6/J or humanized canonical G6PD mice were incubated with 1,2,3-^13^C_3_-glucose for 1 h to determine metabolic fluxes through glycolysis and the pentose phosphate pathway (PPP). *F*, results confirmed significant decreases in the labeled levels of oxidative phase metabolites of the PPP (^13^C_3_-phosphogluconate and ^13^C_2_-ribose-phosphate) in A- and Med-mice, which corresponded to increases in the ratios of labeled ^13^C_3_-pyruvate/lactate. G6PD, glucose 6-phosphate dehydrogenase; RBC, red blood cell.

Pyruvate levels were found to be inversely proportional to G6PD activity in fresh RBCs from an independent cohort of G6PD-deficient (n = 10) and -sufficient (n = 27) blood donors (Fig. 7, *A–C*). The differences in pyruvate levels between the two groups were exacerbated during blood bank storage up to 42 days (Fig. 7*D*). Similarly, RBCs from G6PD-deficient mice (African A- and Mediterranean variant—Med-) and WT C57BL6/J or humanized canonical G6PD mice (Fig. 7*E*) were incubated with 1,2,3-^13^C_3_-glucose for 1 h to determine metabolic fluxes through glycolysis and the pentose phosphate pathway (PPP). Results (Fig. 7*F*) confirmed significant decreases in the labeled levels of oxidative phase metabolites of the PPP (^13^C_3_-phosphogluconate and ^13^C_2_-ribose-phosphate) in A- and Med-mice, which corresponded to increases in the ratios of labeled ^13^C_3_-pyruvate/lactate.

## Discussion

As part of the REDS-III RBC-Omics study, a cohort of 12,535 volunteer blood donors were enrolled to donate a unit of blood that was processed into RBC components that were characterized for storage hemolysis parameters. DNA samples derived from donation WBC were genotyped using a precision transfusion medicine array mapping 879,000 SNPs (30). Phenotypes that were associated with these polymorphisms related to RBC propensity to hemolyze, either spontaneously or following oxidant or osmotic insults (28). As a result, 27 loci were associated with measures of hemolysis following blood storage, the most significant being the association between ANK and SPTA1 with osmotic hemolysis (5.85 × 10^−28^ and 1.01 × 10^−22^, respectively) and G6PD with oxidative hemolysis (2.66 × 10^−17^) (28). Here, we performed the first mQTL analysis of RBCs from 250 recalled RBC-Omics donors, a subset of the 12,535 enrolled and genotyped donors in the RBC-Omics study.

Overall, we report 2831 SNP-metabolite associations meeting genome-wide significance. Of note, the smallest *p*-value found in the present study, *p* = 1.90 × 10^−63^ for the association between rs4539242 within *PYROXD2* and the RBC levels of N-methyl-lysine, was much smaller than the *p*-values describing any of the 27 loci associated with RBC hemolysis phenotypes in this population (citation 28), despite the much smaller cohort (250 *versus* 12,535)—suggesting the metabolic signatures are more directly determined by genetics than is hemolytic propensity, with the latter having a larger etiologic contribution from environmental factors. The association between *PYROXD2* and methyl-lysine had already been reported in previous mQTL studies (40, 41, 52, 53, 54, 55) and thus serves as an internal control for the present analysis.

Novel findings include the association between polymorphisms in the heme transporter FLVCR1 and the RBC levels of choline. Previous genomics data have shown a strong linkage (co-dependency: 0.41 Pearson) between FLVCR1 and the enzyme choline kinase A (https://depmap.org/portal/gene/CHKA?tab=overview). By controlling intracellular heme pools, FLVCR1 is known to play a role in the differentiation of committed erythroid progenitors (56). Like methionine, choline is a methyl-group donor to recharge SAM, and thus, it indirectly participates in nucleotide synthesis during erythropoiesis (57) and methylation events to repair isoaspartyl-damage upon oxidant insults *in vivo* and *in vitro* (58). In this view, it is interesting to note that SAM levels were associated with polymorphisms in the structural protein SPTA1, recently identified as one of the main targets of methylation of deamidated asparagines in stored RBCs (59). The association between choline and FLVCR1 is also relevant owing to the role of choline metabolism as a substrate for phosphocholine metabolism in phospholipid synthesis in terminal erythropoiesis (60).

The RBC levels of multiple lysophosphatidylserines (LPS 16:0, 18:0, 18:1, and 18:2) were associated with variation in the lysophosphatidylcholine acetyltransferase 5 gene (LPCAT3). LPCAT3 is a key enzyme of the Lands cycle (61), which participates in the repair of oxidatively damaged lipids, including LPS, and the secretion of triglycerides through regulation of arachidonoyl-phospholipid metabolism in RBCs (62). These results are suggestive of a role of LPCAT3 in phosphatidylserine metabolism, a class of lipids whose exposure in the outer membrane leaflet regulates erythrophagocytosis and clearance from the bloodstream, with implications for posttransfusion recovery of stored RBCs (10). In this view, it is worth noting that the levels of multiple acyl-carnitines, in equilibrium with the acyl-CoAs as part of the Lands cycle, were found to be associated with polymorphisms in the carnitine transporter SLC22A16. This observation may indicate an intersubject variability in membrane lipid damage–repair capacity, with implications for exercise physiology or kidney disease, since this pathway is impacted by acute exercise (43) or (hypoxia-induced) kidney dysfunction (51) and carnitine-containing supplements. It is worth noting that polymorphic TOP1MT, which limits doxorubicin-induced cardiotoxicity (63) and kidney dysfunction (50), was previously associated with interdonor variability in the levels of methionine—suggestive of a potential axis in RBC oxidant stress damage–repair mechanisms for proteins (58) and lipids (50, 64). Similarly, this observation may be relevant to storage quality and the implementation of carnitine-containing blood storage additives (65). Heterogeneity in the RBC levels of some (bacteria or under sterile *ex vivo* conditions, oxidant-stress derived) odd chain acyl-carnitines (*e.g.*, undecanoyl-carnitine) was associated with polymorphisms in the coding region of the gene EPHX2, which was in turn also associated with variance in the levels of several linoleyl-derived oxylipins (9,10-EpOME, 12,13-EpOME), which are lipid mediators released by RBCs in response to hypoxia (66). It is worth noting that the levels of arachidonic acid were here found to association with polymorphisms in fatty acid desaturase 1, confirming prior findings (67). This is relevant in that mature RBCs have been found to express functional fatty acid desaturases, and fatty acid desaturases activity—which is dependent on iron—was found to increase in response to storage-induced or pathological oxidant stress *in vitro* and *in vivo* (68). Prior work in mice found an association between the levels of oxylipins, iron metabolism (the ferrireductase STEAP3), and poor posttransfusion recovery of stored RBCs (69). Of note, another iron-dependent enzyme (70) spermine oxidase was found to be polymorphic in routine blood donors, which was here associated with varying levels of the product of its enzymatic activity, the polyamine spermine.

One of the main findings of the genomic arm of the REDS-III RBC-Omics Study was the identification of polymorphisms associated with the expression of a less active isoform of G6PD (African variant) that are associated with an increased susceptibility to end of storage hemolysis of RBCs following oxidant insults (28). Parallel metabolomics studies identified an impact for donor sex, ethnicity, and age on the antioxidant systems (especially glutathione-dependent systems) of stored RBCs, with an emphasis for an impairment in the stored RBC capacity to activate the pentose phosphate pathway (16) (G6PD is the rate-limiting enzyme of this pathway). These results were independently corroborated by the observation that failure to activate the PPP is a hallmark of the metabolic lesion to stored RBCs, in part attributable to the inability to inhibit glycolysis *via* the reversible oxidation of glyceraldehyde 3-phosphate dehydrogenase (GAPDH) (71) and loss of GAPDH binding to the N-terminus cytosolic domain of band 3 (72, 73), owing to fragmentation of the latter, as mediated by caspase activity or oxidant stress (74). Here, we report that the levels of pyruvate in fresh RBCs and pyruvate/lactate ratios in stored RBCs are associated with the same G6PD polymorphisms. A causal role of this correlation is established in humanized murine models of G6PD deficiency, since the same metabolic change is seen in RBCs that differ only in their form of G6PD (African or Mediterranean (75) variants *versus* nondeficient human form). These observations could be partly explained by the compensatory overactivation of NADH-dependent methemoglobin reductase to cope with increased oxidant stress in G6PD deficient erythrocytes (31). Indeed, methemoglobin reductase would compete with lactate dehydrogenase for NADH, rendering the enzymatic step of lactic fermentation to regenerate NADH back to NAD+ no longer critical to preserve glycolytic fluxes (NAD+ is an essential cofactor for GAPDH activity upstream to pyruvate and ATP synthesis in glycolysis). The G6PD African variant in this donor population was also linked to variance in the levels of dopamine, confirming previous biomarker analyses from the metabolome of G6PD-deficient *versus* sufficient blood donors (16). This is interesting in that monoamine oxidase-dependent dopamine synthesis is an NADPH-dependent process, with implications relevant to exercise physiology (*e.g.*, the sense of well-being/stimulatory effect after exercise (76)).

The present study has several limitations. First, mQTL analyses were determined based upon genomic characterization of a cohort of volunteer routine blood donors. As such, disease-related polymorphisms that would be identified in cohorts of nonhealthy patients (*i.e.*, from persons who are ineligible to donate blood) would be intrinsically not amenable to identification as a result of our study design. On the other hand, while sufficiently healthy to donate blood and as a result probably has biases similar to other ‘healthy worker’ cohorts (77), the donor population enrolled in this study also includes phenotypes of potential clinical relevance to disease phenotypes (*e.g*., to cardiovascular and other disease risk factors, such as obesity (27), smoking (23), and alcohol consumption (25)) but at a lower rate than the general population. As such, some of the genome-wide associations reported here (*e.g.*, carnitine and SLC22A16) may be translationally relevant beyond transfusion medicine when interpreted in the context of markers relevant to specific diseases (*e.g.*, carnitine metabolism and obesity (27)). Furthermore, fresh (∼10 day old—*i.e.*, freshest samples available for this cohort) RBCs from volunteer donor volunteers were tested in this study. As such, it is unclear whether the findings are relevant to transfusion medicine (*e.g.*, genetic underpinning of metabolic heterogeneity in end of storage RBCs) or to physiological (*e.g.*, hypoxia) or pathological conditions in which alterations to RBC metabolism are mechanistically relevant. Indeed, some metabolic markers of the RBC storage lesion only accumulate in end of storage units (*e.g.*, hypoxanthine) (35). However, replication studies were performed on end of storage (day 42) blood from the same units and donors, though only 176 biological replicates were available. As such, the present findings are suggestive of clinical relevance in the field of transfusion medicine, to the extent that the metabolic heterogeneity of fresh and end of stored units associates or is an etiological driver of posttransfusion performances, such as intravascular or extravascular hemolysis and posttransfusion recoveries (10, 16, 17, 18). Future studies will need to address this issue in larger cohorts, by focusing on RBC samples stored for longer periods of time. Although the small (n = 250) number of participants available still allowed for robust association discovery, a larger number of samples in more ancestrally diverse participants will increase the statistical power of future work and provide insights that are relevant to specific populations. Such studies could pave the way for the use of other orthogonal omics approaches to metabolomics (*e.g.*, proteomics) to maximize the value of the genetic and metabolic data already available for this well-curated cohort. Similar studies could be possible on other cohorts from patients with hematological conditions, such as sickle cell disease, where metabolite levels could not only be associated with but also mechanistically contribute to the etiology of thromboinflammatory comorbidities of clinical relevance (*e.g.*, sphingosine 1-phosphate and systemic hypoxemia (78), vaso-occlusive crisis, cardiopulmonary function, kidney dysfunction, pain crisis, *etc*.).

## Experimental procedures

### REDS-III RBC-Omics study participants and samples

RBC-Omics was conducted under regulations applicable to all human subject research supported by federal agencies as well as requirements for blood product manipulation specified and approved by the FDA. The data coordinating center (RTI International) of REDS-III was responsible for the overall compliance of human subjects regulatory protocols including institutional review board approval from each participating blood center, from the REDS-III Central Laboratory (Vitalant Research Institute), and from the data coordinating center, as previously detailed (16, 29). Donors were enrolled at the four participating REDS-III US blood centers. Overall, 13,758 whole blood donors were enrolled, and 13,403 (97%) age 18+ provided informed consent to participate in the study; of these, 12,353 were evaluated for hemolysis parameters (spontaneous, oxidative, or osmotic) on RBCs stored for ∼39 to 42 days. Extreme hemolyzers (5th and 95th percentile) from the donors tested for end of storage oxidative hemolysis were asked to donate a second unit of blood. These units were sterilely sampled for metabolomics analyses (n = 250 for the freshest available time points, *i.e*., < 14 storage days). Blood collection, sample processing, and other aspects of the screening and recall phases of the RBC-Omics Study have been extensively described (26, 28).

### Sample processing and metabolite extraction

An isotopically labeled internal standard mixture, including a mix of ^13^C^15^N-labeled amino acid standards (2.5 μM), was prepared in methanol. A volume of 100 μl of frozen RBC aliquots was mixed with water and the mixture of isotopically labeled internal standards (1:1:1, *v/v/v*). The samples were extracted with methanol (final concentration of 80% methanol). After incubation at −20 °C for 1 h, the supernatants were separated by centrifugation and stored at −80 °C until analysis. Samples were vortexed and insoluble material pelleted as described (16, 29).

### Ultra-high-pressure liquid chromatography-mass spectrometry metabolomics

Analyses were performed using a Vanquish UHPLC coupled online to a Q Exactive mass spectrometer (Thermo Fisher). Samples were analyzed using a 3 min isocratic condition or a 5, 9 and 17 min gradient as described (79, 80, 81). Solvents were supplemented with 0.1% formic acid for positive mode runs and 1 mM ammonium acetate for negative mode runs. MS acquisition, data analysis, and elaboration was performed as described (79, 80, 81). Additional analyses, including untargeted analyses and Fish score calculation *via* MS/MS, were calculated against the ChemSpider database with Compound Discoverer 2.0 (Thermo Fisher).

### Metabolite QC and processing

The quality control and processing of metabolites is detailed in Fig. S1. We first selected only those metabolites measured at day 10 of storage, for which 250 participants had metabolite data. Metabolites with missing data and zeros were both treated identically as missing. We removed the following metabolites from further processing: a duplicate carnosine, a duplicate lorazepam, phosphate, triacanthine, and acetyl-L-carnitine. Five hundred seven metabolites remained for further processing. We also removed 22 drug metabolites with concentrations detected in greater than 50% of the participants, leaving 487 metabolites. We then separated the participant data by blood storage additive type and excluded metabolites with greater than 10% missingness from each additive set, respectively. After removing these metabolites, 382 remained. We separated these 382 metabolites into those quantified absolutely (against stable isotope-labeled internal standards, as described (29)) *versus* relatively. Relatively quantified metabolites were natural log transformed. A suffix “(uM)” was added to the label of all the metabolites for which absolute concentrations were determined. These groups of metabolites then had missing metabolite levels imputed using QRILC (82), implemented in the R package QRILC (82). After imputation, all metabolites were inverse-normal transformed using the R package GenABEL rntransform command (83).

### Genotyping and imputation

Details of the genotyping and imputation of the RBC-Omics study participants have been previously described by Page *et al*. (28) Briefly, genotyping was performed using a transfusion medicine microarray (30) and the data are available in dbGAP accession number phs001955.v1.p1. Imputation was performed using 811,782 SNPs that passed quality control. After phasing using Shape-IT (84), imputation was performed using Impute2 (85) with the 1000 Genomes Project phase 3 (85) all-ancestry reference haplotypes. We used the R package SNPRelate (86) to calculate principal components of ancestry.

### Genome-wide association study

We performed association analyses for each of the 382 metabolites using an additive SNP model in the R package ProbABEL (87) and 243 study participants who had both metabolomics data and imputation data on serial samples from stored RBC components that passed respective quality control procedures. We adjusted for sex, age (continuous), frequency of blood donation in the last 2 years (continuous), blood donor center, and 10 ancestry principal components. Statistical significance was determined using a *p*-value threshold of 5 x 10^-8^. We only considered variants with a minimum minor allele frequency of 1% and a minimum imputation quality score of 0.80.

### Replication and sensitivity analyses

For replication, we followed the same procedures for postprocessing of metabolites measured at day 42 of storage. There were 176 participants with metabolite data generated from day 42 samples. Association analyses and statistical significance were determined as described above. We selected 46 metabolites, oversampled for top hits from the GWAS analysis of early storage samples to analyze for potential replication and in the sensitivity analyses described below.

We performed four sensitivity analyses using 46 metabolites and the original 243 recalled RBC-Omics participants. We performed a “stringent” GWAS, which required that evaluated variants have a minimum minor allele frequency of 5% and a minimum imputation quality score of 0.90. We performed an analysis using only those participants whose blood donations were collected at one of the three centers that used Additive 3 in their storage protocol. We also performed a sensitivity analysis including only those participants of European ancestry and using the variant data imputed using the European reference panel. Finally, we re-imputed the missing metabolites data as described above, swapping out the QRILC imputation procedure for a simple substitution of the missing value with the lowest detected value for the metabolite in question.

### OASIS queries

The OASIS: Omics Analysis, Search & Information, a TOPMED funded resources (88), was used to annotate the top SNPS. OASIS annotation includes information on position, chromosome, allele frequencies, closest gene, type of variant, position relative to closest gene model, if predicted to functionally consequential, tissues-specific gene expression, and other information.

### LocusZoom plots

We generated LocusZoom plots locally using v1.4 and plotted a margin of ± 200 kilobases around each lead SNP against the November 2014 1000 Genomes European ancestry build.

### Comparison with GWAS catalog

Lead SNPs for all metabolites were queried using the LDLink tool LDtrait (query date 5/6/2022) (89) by selecting an R^2^ threshold of 0.8 in a ± 500,000 base pair window in the combined five European ancestries using Genome Build GRCh37. We noted SNPs that have been previously associated with other traits and considered replicated associations as those SNPs with previously reported associations to the same metabolite as found in our study population.

### Animal models

All animal procedures were approved by the University of Virginia IACUC (protocol no. 4269). Humanized G6PD-deficient (A-, Med-) and nondeficient (huCan) mice were generated by replacing the murine G6PD locus in Bruce4 ES cells (C57BL/6 background) with either the A- (V68M/N126D), Med- (S188F), or huCan (B+) variant (*manuscript in preparation*). In short, nucleofected ES cells were drug selected (Neo), G418-resistant clones were isolated, and the presence of homologous recombination (and absence of random integration) was confirmed (data not shown). Clones were then developed into full animals, and correct homologous recombination was reconfirmed. The Neo cassette was flanked with FRT sites and removed by breeding with a germline FLP transgenic mouse—the FLP was subsequently removed. Generation of Cre-inducible G6PD Med-deficient mice was described previously (75).

### G6PD-deficient subjects

An independent cohort was enrolled in this study at the Columbia University and New York Blood Center in New York, under IRB protocols no. AAAJ6862 and 401,165, respectively. Male volunteers were recruited using flyers, person-to-person communication, and email, between November 2012 and August 2017. Screening was limited to males because G6PD deficiency is X-linked; Following screening and confirming G6PD activity, 10 G6PD-deficient and 30 G6PD-normal males donated 1 unit of whole blood at the New York Blood Center (17), each of which was processed into packed RBCs, leukoreduced, prior to metabolomics analysis.

## Data availability

All the mQTL results and related elaborations described in the present study are provided in Table S1. The raw genomics data were made available as per Page *et al*. J Clin Investigation 2021 (reference (28)). The raw metabolomics data were made available as per D’Alessandro *et al*. Transfusion 2019 (reference (29)).

## Supporting information

This article contains supporting information.

## Conflict of interest

Though unrelated to the contents of this manuscripts, the authors declare that A. D. is a founder of Omix Technologies Inc and Altis Biosciences LLC. A. D. is SAB members for Hemanext Inc. and FORMA Therapeutics Inc. A. D. is a consultant for Rubius Therapeutics. J. C. Z. is a consultant for Rubius Therapeutics and a founder of Svalinn Therapeutics. All other authors have no conflicts of interests to disclose.
