# Supplementary material for: Genome-wide metabolite quantitative trait loci analysis (mQTL) in red blood cells from volunteer blood donors
Source: J Biol Chem. 2022 Nov 15;298(12):102706. doi: 10.1016/j.jbc.2022.102706 (PMC9763692; doi:10.1016/j.jbc.2022.102706)
Supplement: Supplementary figure [file mmc2.pdf]

Supplementary Figures for:

Genome-wide metabolite quantitative trait loci analysis (mQTL) in red blood cells from volunteer blood donors

Amy Moore,[1] Michael P Busch,[2] Karolina Dziewulska,[3] Richard O. Francis,[4] Eldad A. Hod,[4] James C Zimring,[3] Angelo D'Alessandro,[4,#] Grier P Page,[1,*]

1) Division of Biostatistics and Epidemiology, RTI International, Atlanta, GA, USA
2) Vitalant Research Institute, San Francisco, CA, USA
3) Department of Pathology, University of Virginia School of Medicine, Charlottesville, VA, USA
4) Department of Pathology and Cell Biology, Columbia University Medical Center, New York, NY, USA
5) Department of Biochemistry and Molecular Genetics, University of Colorado Denver – Anschutz Medical Campus, Aurora, CO, USA;

***Corresponding authors:**

Grier P Page, PhD
RTI International
Division of Biostatistics and Epidemiology
2987 Clairmont Rd, Suite 400
Atlanta, Georgia 30340, USA.
Phone:# 770.407.4907
E-mail: gpage@rti.org

Angelo D'Alessandro, PhD
Department of Biochemistry and Molecular Genetics
University of Colorado Anschutz Medical Campus
12801 East 17th Ave., Aurora, CO 80045
Phone # 303-724-0096
E-mail: angelo.dalessandro@ucdenver.edu

Running title: *mQTL in RBCs from blood donors*

Supplementary Table 1 – Please refer to the file *Supplementary Table 1.xlsx*

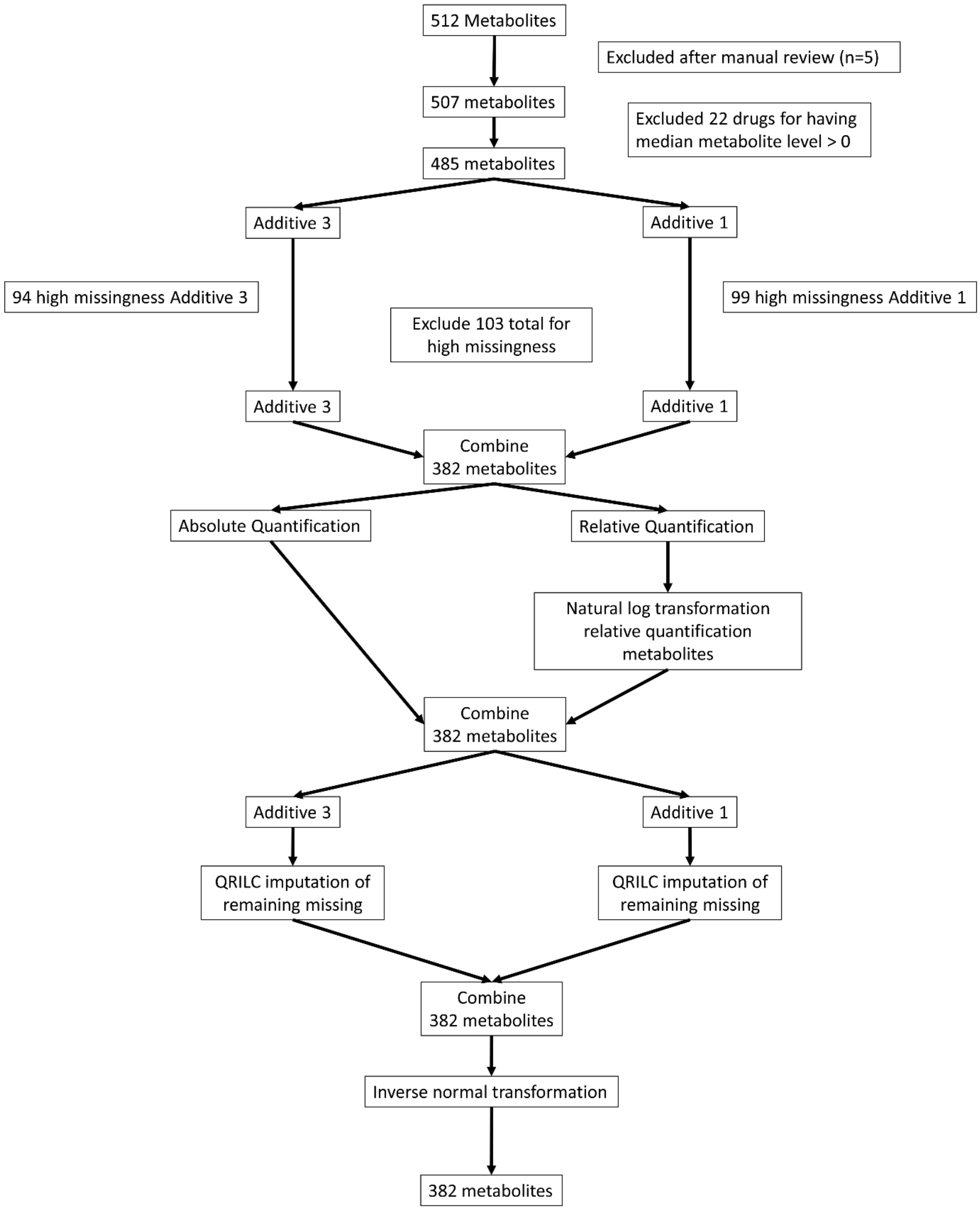

Supplementary Figure 1 – Flow-chart of metabolomics post-processing.

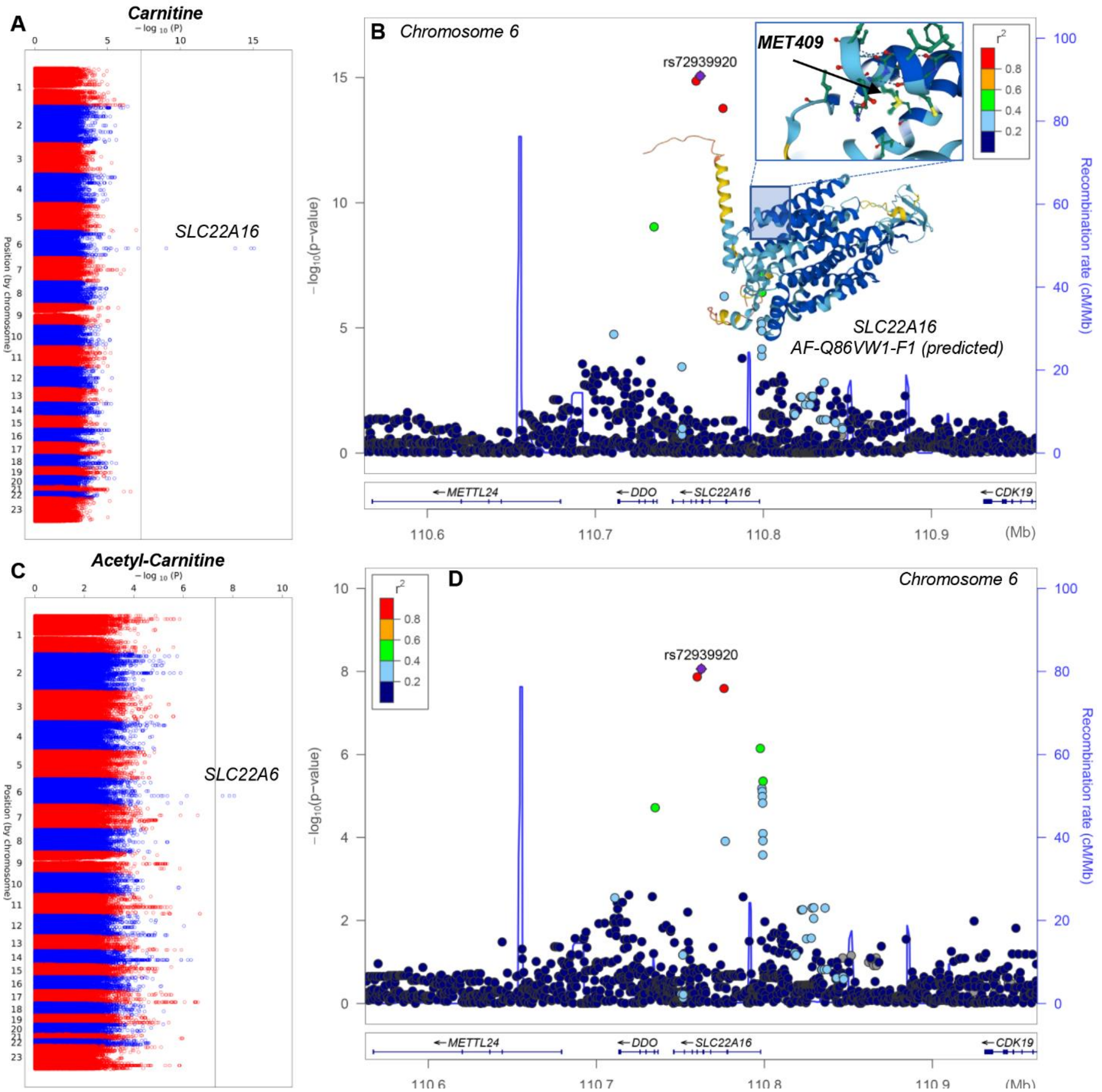

Supplementary Figure 2 – Polymorphisms in the carnitine transporter SLC22A16 are associated with variability in the levels of RBC free and acetyl-carnitine. Manhattan plots and LocusZooms are shown in panels **A-B** and **C-D**, respectively.

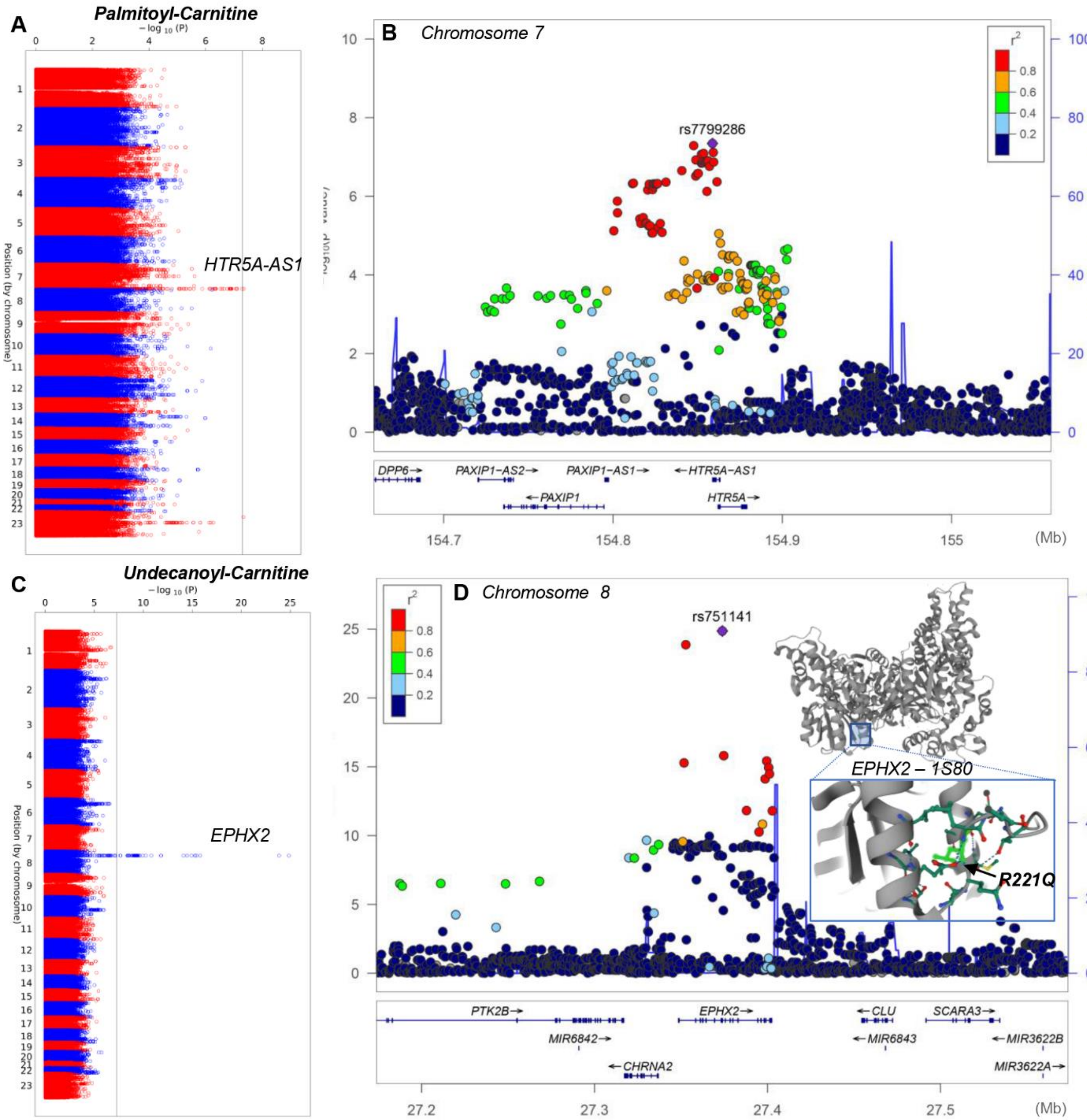

Supplementary Figure 3 – Polymorphisms in HTR5A-AS1 and EPHX2 are associated with variability in the levels of RBC palmitoyl- and undecanoyl-carnitine- an odd-chain fatty acyl-carnitine derived from lipid peroxidation reactions. Manhattan plots and LocusZooms are shown in panels **A-B** and **C-D**, respectively.

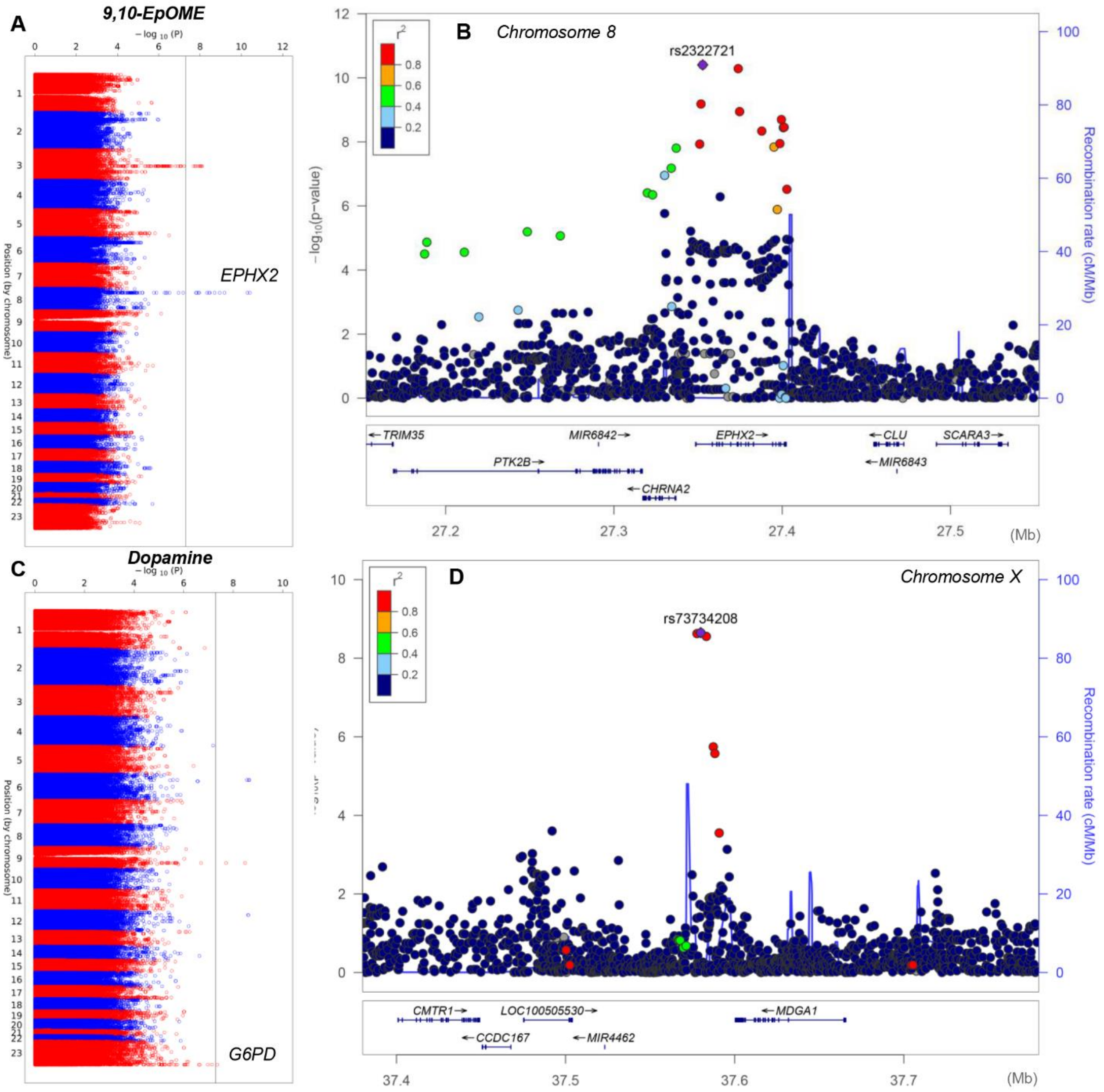

Supplementary Figure 4 – Polymorphisms in the EPHX2 and G6PD are associated with variability in the levels of RBC 9,10,EpOME (oxylipin) and dopamine. Manhattan plots and LocusZooms are shown in panels **A-B** and **C-D**, respectively.

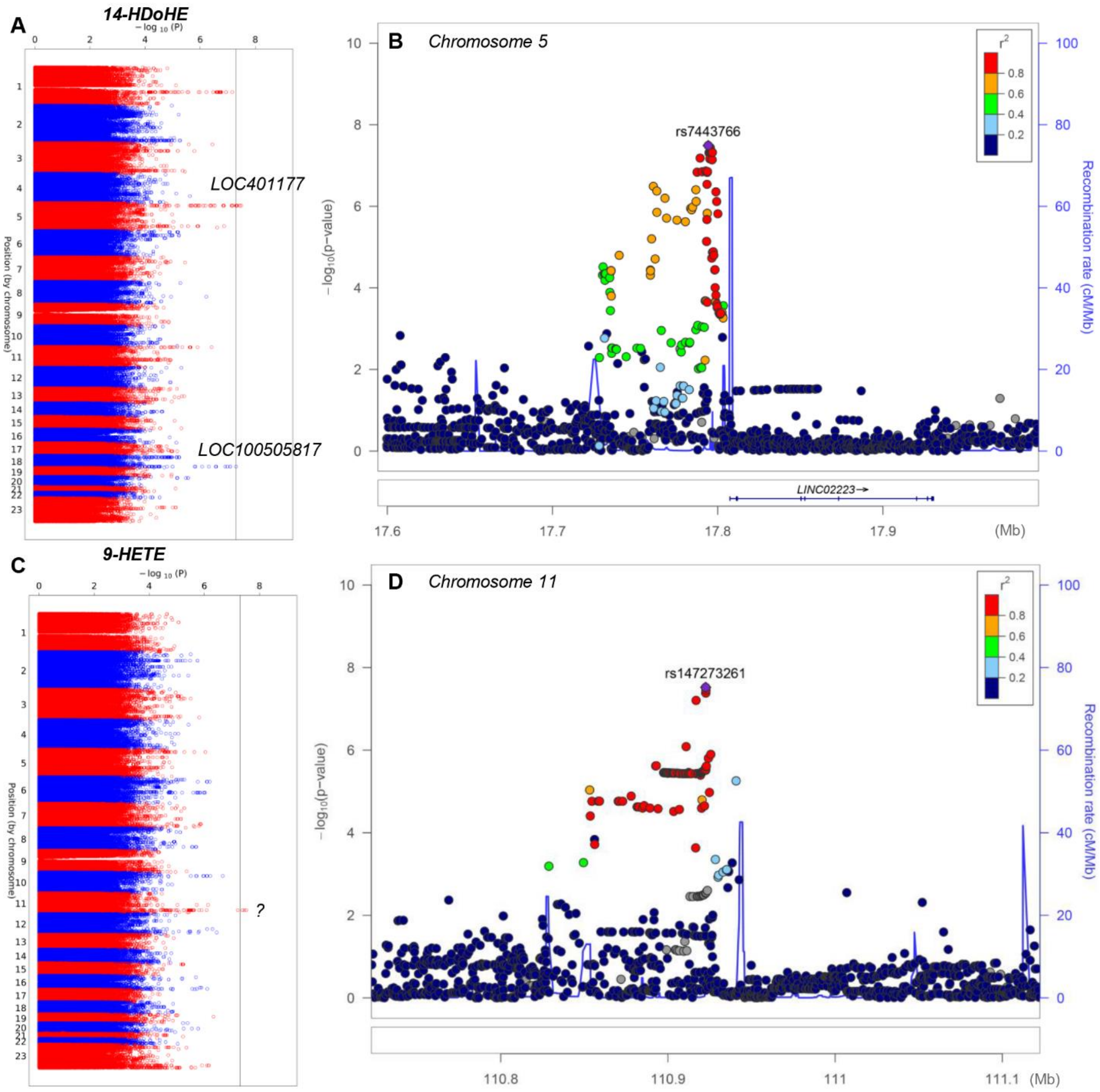

Supplementary Figure 5 – Polymorphisms in LOC401177 and rs1472723261 SNP are associated with variability in the levels of RBC oxylipins 14-HDoHE and 9-HETE. Manhattan plots and LocusZooms are shown in panels **A-B** and **C-D**, respectively.

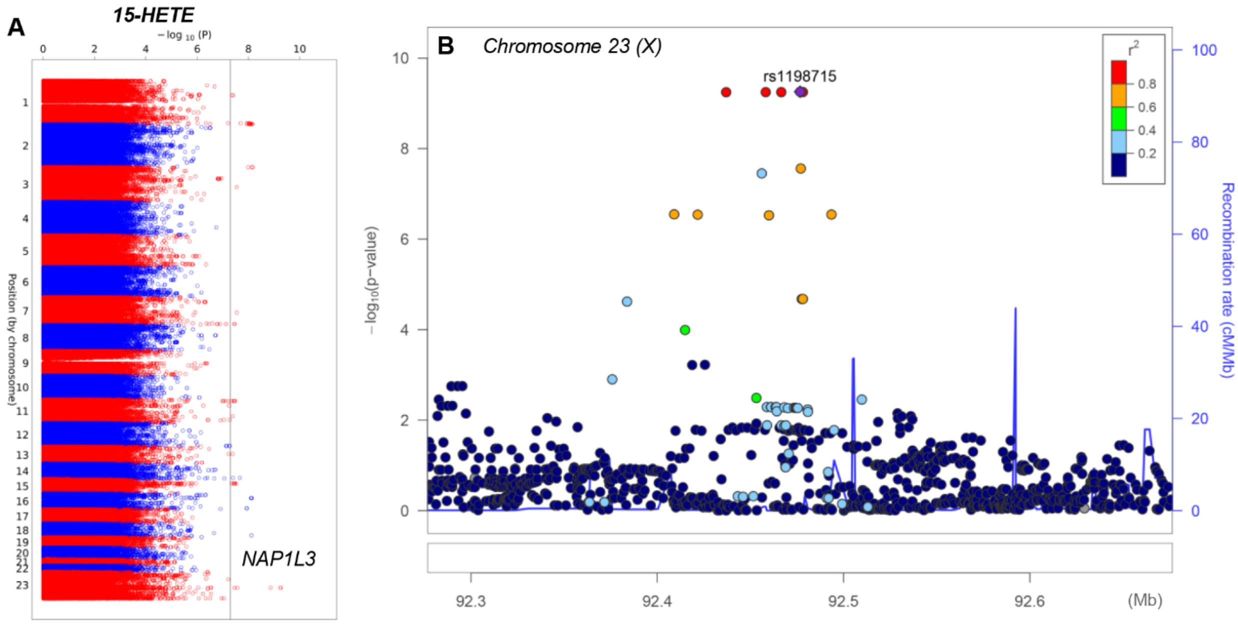

Supplementary Figure 6 – Polymorphisms in the coding region for NAP1L3 are associated with variability in the levels of RBC 15-HETE. Manhattan plots and LocusZooms are shown in panels **A-B** and **C-D**, respectively.

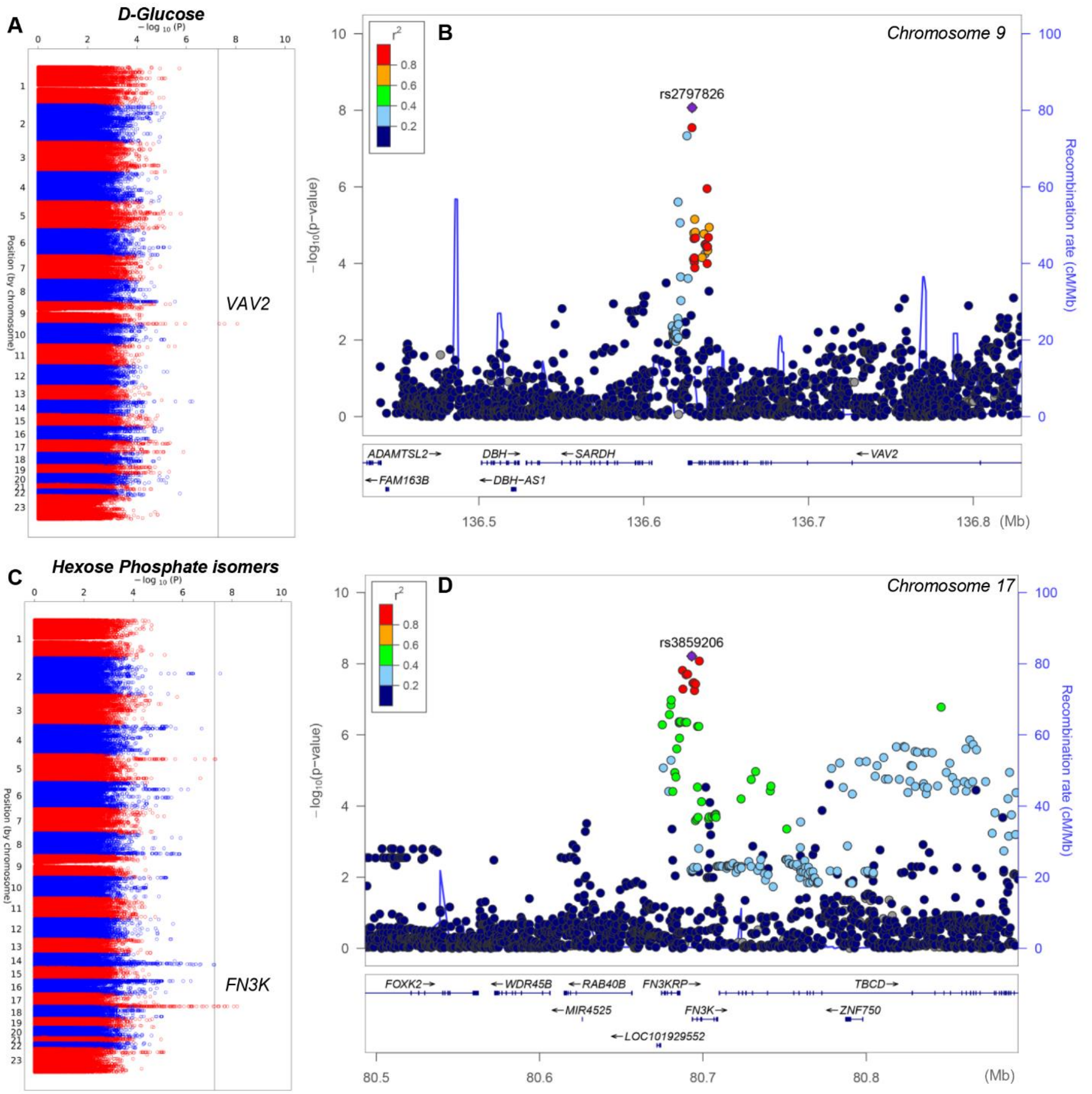

Supplementary Figure 7 – Polymorphisms in VAV2 and FN3K are associated with variability in the levels of RBC glycolytic metabolites glucose and hexose phosphate isomers. Manhattan plots and LocusZooms are shown in panels **A-B** and **C-D**, respectively.

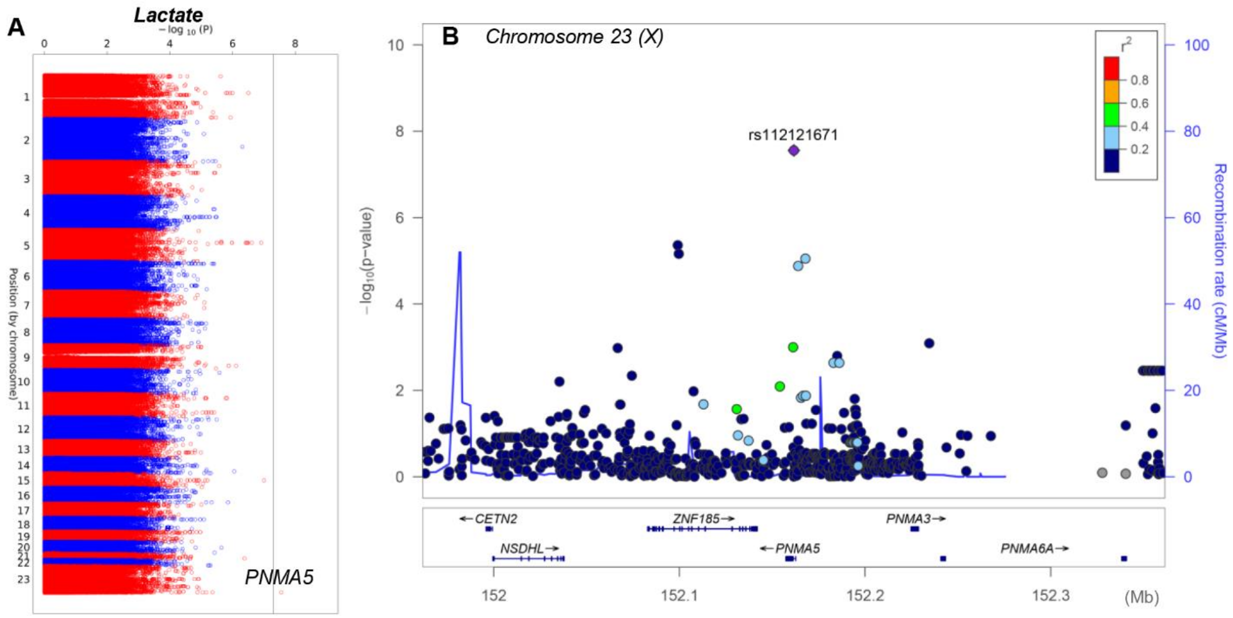

Supplementary Figure 8 – Polymorphisms in PNMA5 are associated with variability in the levels of RBC lactate. Manhattan plots and LocusZooms are shown in panels **A-B**, respectively.

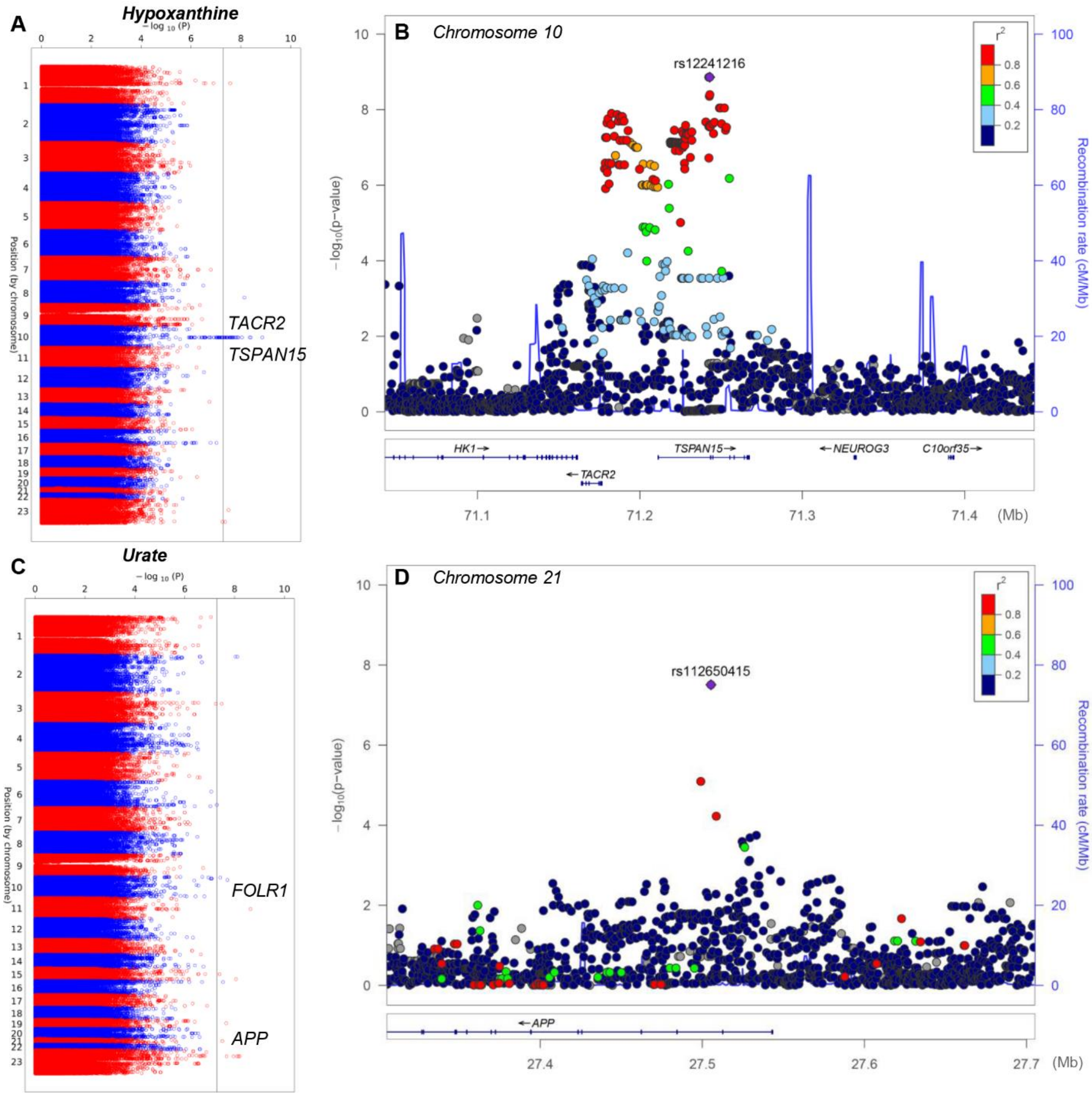

Supplementary Figure 9 – Polymorphisms in TACR2 or TSPAN15 and APP or FOLR1 are associated with variability in the levels of RBC purine deamination products hypoxanthine and urate. Manhattan plots and LocusZooms are shown in panels **A-B** and **C-D**, respectively.

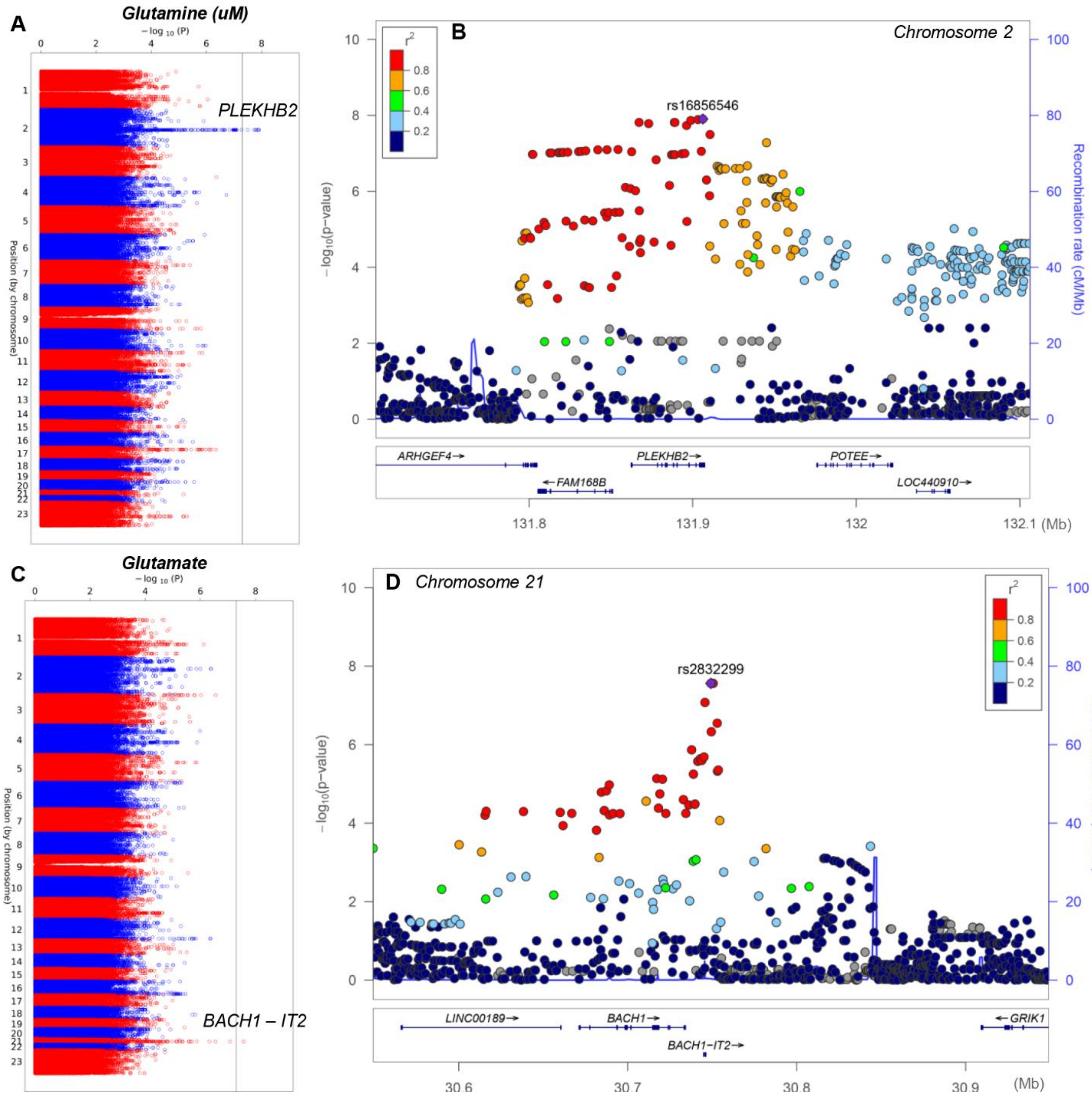

Supplementary Figure 10 – Polymorphisms in PLEKHB2 and BACH1-IT2 are associated with variability in the levels of RBC amino acids glutamine and glutamate. Manhattan plots and LocusZooms are shown in panels **A-B** and **C-D**, respectively.

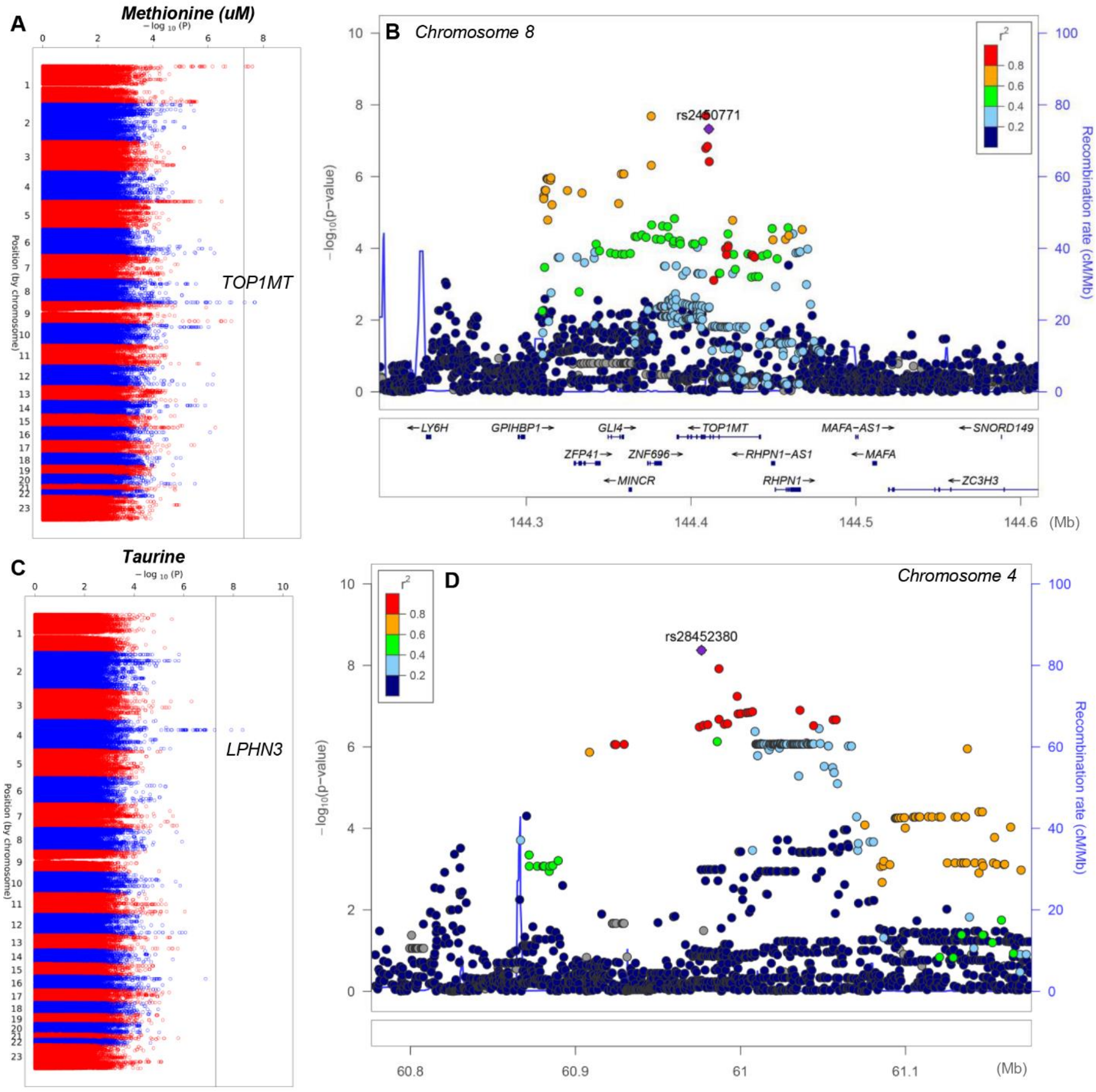

Supplementary Figure 11 – Polymorphisms in TOP1MT and LPHN3 are associated with variability in the levels of RBC amino acids methionine and taurine. Manhattan plots and LocusZooms are shown in panels **A-B** and **C-D**, respectively.

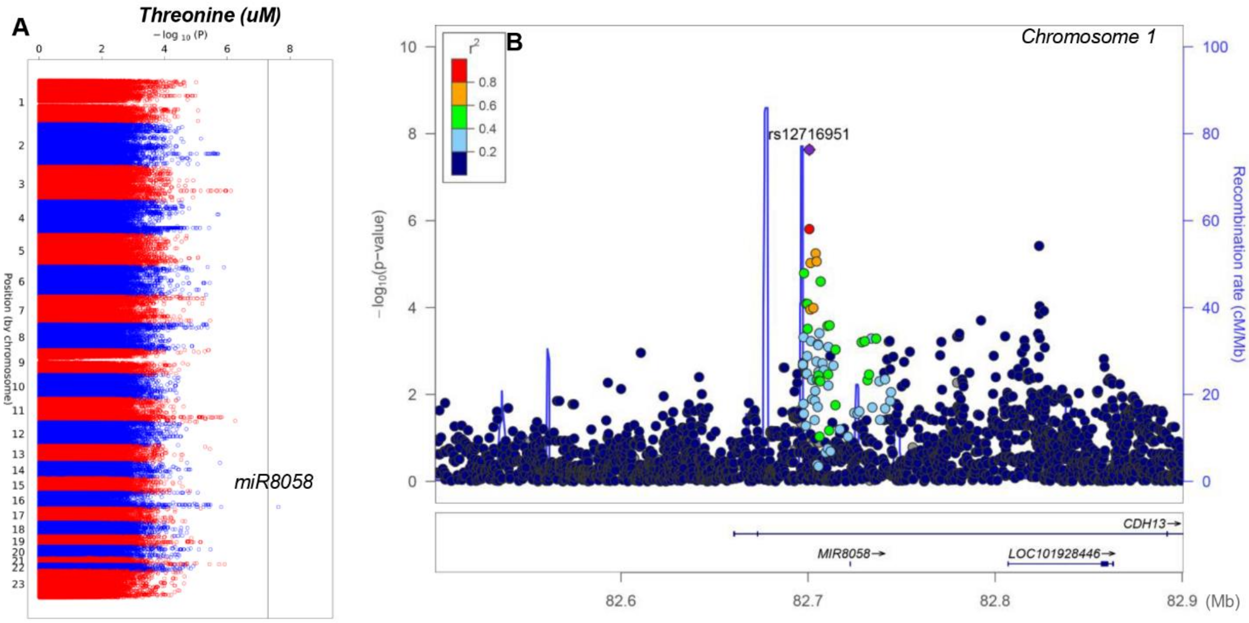

Supplementary Figure 12 – Polymorphisms in miR8058 are associated with variability in the levels of RBC threonine. Manhattan plots and LocusZooms are shown in panels **A-B**, respectively.

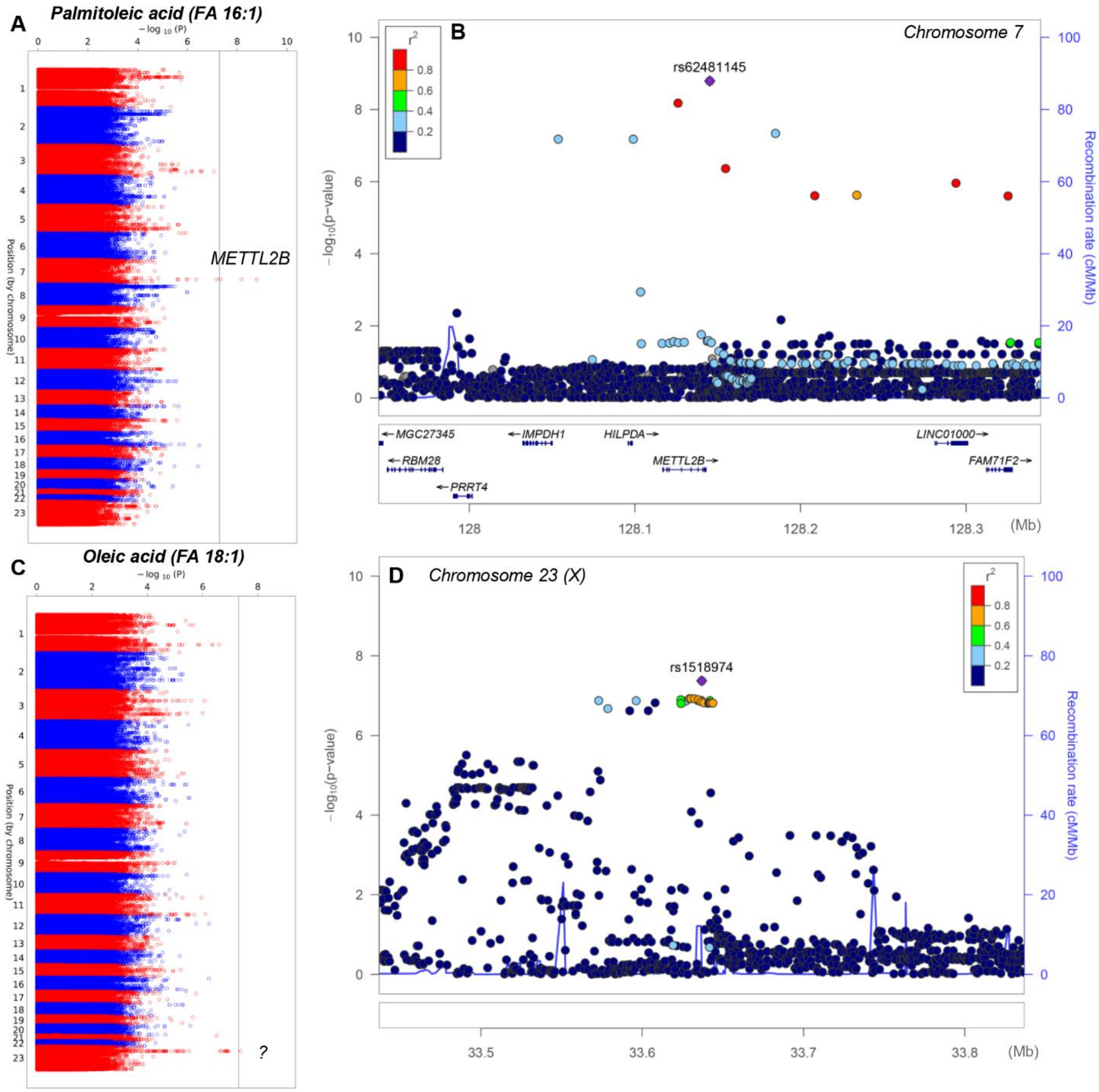

Supplementary Figure 13 – Polymorphisms in METTL2b and rs1518974 SNP are associated with variability in the levels of RBC monounsaturated fatty acids palimitoleic and oleic acid. Manhattan plots and LocusZooms are shown in panels **A-B** and **C-D**, respectively.

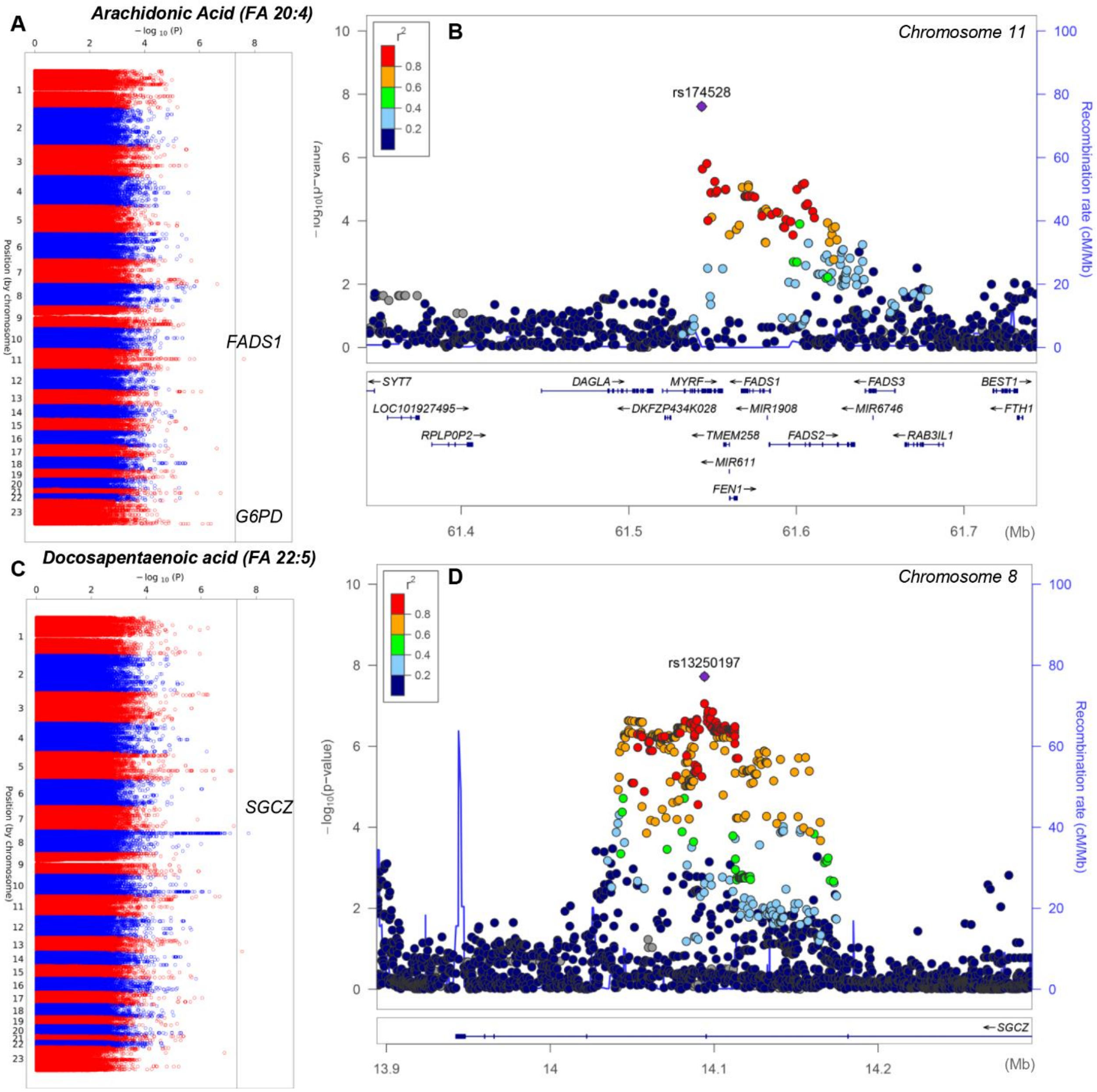

Supplementary Figure 14 – Polymorphisms in FADS1 or G6PD and SGCZ are associated with variability in the levels of RBC highly-unsaturated fatty acids arachidonate and docosapentanoate. Manhattan plots and LocusZooms are shown in panels **A-B** and **C-D**, respectively.

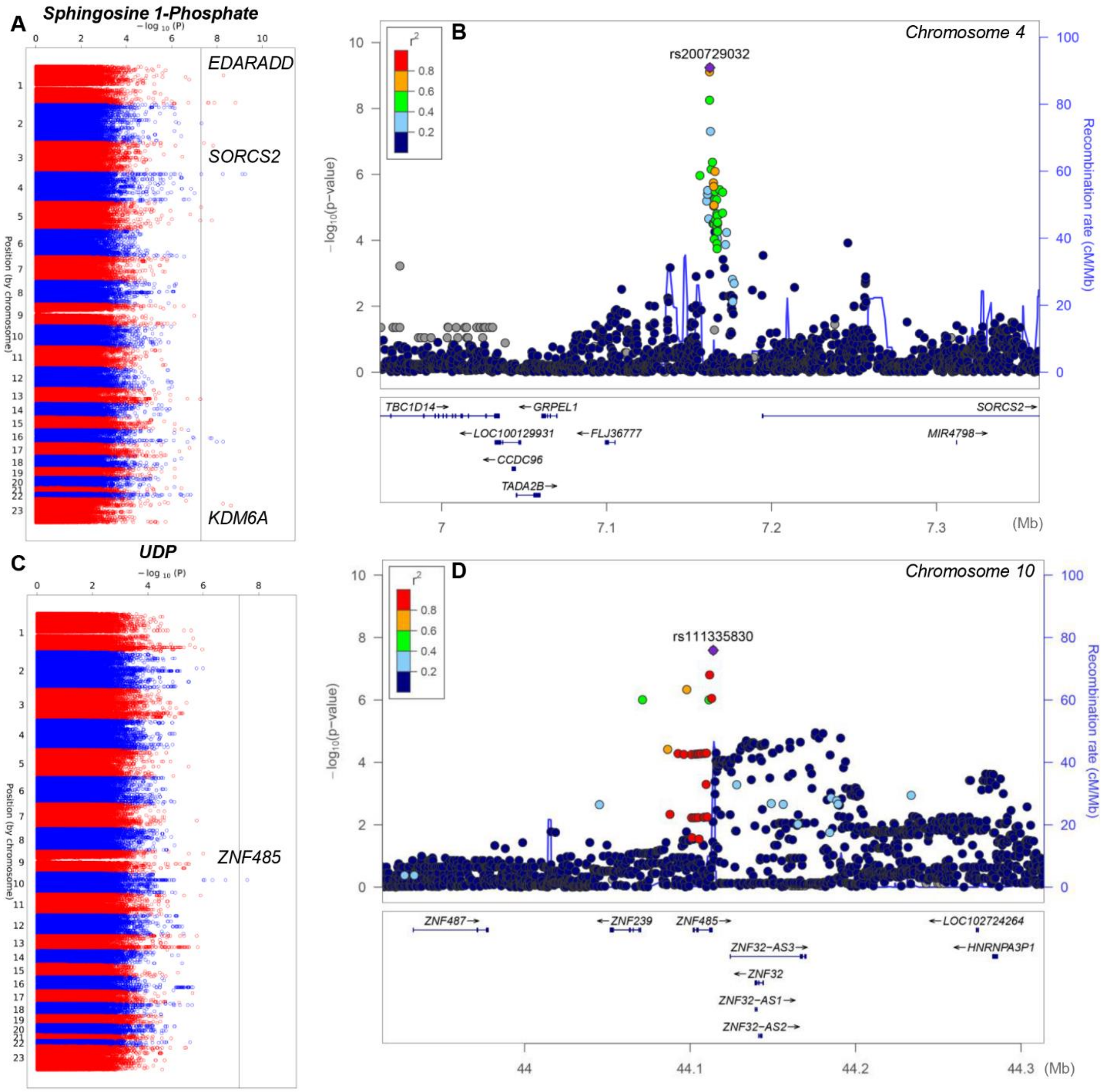

Supplementary Figure 15 – Polymorphisms in EDARADD, SORCS2 or KDM6A and ZNF485 are associated with variability in the levels of RBC sphingosine 1-phosphate and UDP, respectively**.** Manhattan plots and LocusZooms are shown in panels **A-B** and **C-D**, respectively.
